# Robustness and resilience of computational deconvolution methods for bulk RNA sequencing data

**DOI:** 10.1093/bib/bbaf264

**Published:** 2025-06-12

**Authors:** Su Xu, Duan Chen, Xue Wang, Shaoyu Li

**Affiliations:** Department of Mathematics and Statistics, University of North Carolina at Charlotte, 9201 University City Blvd, Charlotte, NC 28223, United States; Department of Mathematics and Statistics, University of North Carolina at Charlotte, 9201 University City Blvd, Charlotte, NC 28223, United States; School of Data Science, University of North Carolina at Charlotte, 9201 University City Blvd, Charlotte, NC 28223, United States; Department of Quantitative Health Sciences, Mayo Clinic, 4500 San Pablo Rd. S., Jacksonville, FL 32224, United States; Department of Mathematics and Statistics, University of North Carolina at Charlotte, 9201 University City Blvd, Charlotte, NC 28223, United States; School of Data Science, University of North Carolina at Charlotte, 9201 University City Blvd, Charlotte, NC 28223, United States

**Keywords:** deconvolution, robustness, resilience, cellular composition

## Abstract

This study benchmarks the robustness and resilience of computational deconvolution methods for estimating cell-type proportions in bulk tissues, with a focus on comparing reference-based and reference-free methods. Robustness is evaluated by generating *in silico* pseudo-bulk tissue RNA sequencing data from cell-level gene expression profiles derived from four different tissue types, with simulated cellular composition at varying levels of heterogeneity. To assess resilience, we intentionally alter single-cell RNA profiles to create pseudo-bulk tissue RNA-seq data. Deconvolution estimates are compared with ground truth using Pearson’s correlation coefficient, root mean squared deviation, and mean absolute deviation. The results show that reference-based methods are more robust when reliable reference data are available, whereas reference-free methods excel in scenarios lacking suitable reference data. Furthermore, variations in cell-level transcriptomic profiles and cell composition have emerged as critical factors influencing the performance of deconvolution methods. This study provides significant insights into the factors affecting bulk tissue deconvolution performance, which are essential for guiding users and advancing the development of more powerful and reliable algorithms in the future.

## Introduction

The heterogeneity of the cellular composition, i.e. the variation in the cell-type distribution of the bulk tissue samples across different experimental conditions or treatment groups, is frequently observed in biological specimens, due to biological or technical factors. For example, the brains of patients with Alzheimer’s disease (AD), a complex chronic neurodegenerative disease, exhibit significant neuronal loss along with proliferation of microglia and astrocytes [1–3]. Therefore, the cellular composition of AD brain tissue samples is notably different from that of healthy brain tissue samples [4–6]. Another example is pancreatic samples from patients with type 2 diabetes (T2D), which is believed to have significantly fewer $\beta $ cells compared with samples from healthy individuals [7, 8]. In addition, bulk tissue samples taken from different anatomic locations or at different stages of development of a life cycle show varied cell compositions [9]. The heterogeneous cellular composition poses challenges in bulk tissue RNA sequencing (RNA-seq) data analysis, such as differential expression (DE) and gene coexpression network analysis. To address these challenges, computational methods, including reference-based and reference-free algorithms, have been developed to deconvolute bulk tissue RNA-seq data for cell compositions.

Reference-based methods for bulk RNA-seq deconvolution utilize single-cell or purified cell-type expression profiles as references to estimate the proportions of different cell types in bulk tissue samples. These methods apply various statistical and machine learning techniques to achieve accurate deconvolution. Digital Sorting Algorithm (DSA) [10] uses linear regression model and the expression profiles of cell-type-specific marker genes to estimate cell proportions. DeconRNASeq [11] and DCQ [12] employ regression-based approaches, while Bseq-SC [13] uses $\nu $-SVR and enhances estimation using single-cell RNA sequencing (scRNA-seq) data as references. TIMER [14] and EPIC [15] are designed for tumor microenvironment deconvolution, integrating additional biological priors. CIBERSORTx [16] extends CIBERSORT [17] by incorporating single-cell data for improved precision and provides high-resolution cell-type-specific gene expression estimates. dtangle [18] focuses on minimizing bias through carefully selected marker genes by using a linear mixing model. MuSiC [19] leverages cross-subject scRNA-seq data for robust estimations. BayesPrism [20] applies Bayesian modeling to improve inference accuracy. And DAISM-DNN [21] integrates deep learning techniques for enhanced performance. Together, these methods provide diverse strategies for addressing the complexity of bulk RNA-seq deconvolution.

Reference-free deconvolution methods, on the other hand, do not require external reference datasets. These methods rely on matrix factorization, statistical modeling, and optimization techniques to infer cell-type proportions. CellDistinguisher [22] employs a cluster-based approach to identify marker genes for each cell type and then uses support vector machine to separate gene expression signals into distinct cell-type components. TOols for the Analysis of heterogeneouS Tissues (TOAST) [23] improves reference-free deconvolution, which iteratively searches for cell-type-specific features and performs composition estimation. Complete Deconvolution for Sequencing data (CDSeq) [24] implements nonnegative matrix factorization (NMF) to simultaneously estimate cell proportions and cell-type-specific gene expression levels. Linseed [25] uses convex geometry principles to estimate cell-type proportion by identifying expreme points in the expression space and then solves the deconvolution problem via convex optimization techniques. GS-NMF [26] extends NMF by incorporating geometric and solvobility penalty terms to improve interpretability and accuracy. These methods offer flexible options when reference profiles are unavailable. A complete list of these methods are in Table 1.

**Table 1:** An overview of deconvolution algorithms and characteristics

**Method**	**Mathematical/Statistical Foundations**	**Input**	**Output**	**Reference**
REFERENCE-BASED				
DSA	Regularized linear regression	bulk RNA-seq Data and marker genes	cell-type fractions without annotation	[10]
DeconRNASeq	Nonnegative least squares	mRNA-Seq	cell-type fractions	[11]
DCQ	Regularized constrained least squares	reference signatures	Cell-type fractions	[12]
Bseq-SC	$\nu $ -Support Vector Regression	use of scRNA-seq for reference signatures	cell-type fractions	[13]
TIMER	Regularized linear regression (multivariate normal)	reference signatures	cell-type fractions of immune cell types	[14]
EPIC	Weighted constrained least squares	scRNA-seq	cell-type fractions and *in silico* purification (high resolution)	[15]
CIBERSORTx	$\nu $ -Support Vector Regression	use of scRNA-seq for reference signatures	cell-type fractions and *in silico* purification (high resolution)	[16]
dtangle	Linear mixing model of linear-scale expressions	scRNA-seq and marker genes	cell-type fractions	[18]
MuSiC	Weighted least squares	scRNA-seq	cell-type fractions	[19]
BayesPrism	Bayesian model	scRNA-seq	cell-type fractions	[20]
DAISM-DNN	Deep neural network	scRNA-seq and bulk RNA profile	cell-type fractions	[21]
REFERENCE-FREE				
CellDistinguisher	Nonnegative matrix factorization	bulk RNA-seq Data	cell-type fractions without annotation	[22]
TOAST	Nonnegative matrix factorization and principal component analysis	bulk RNA-seq Data	cell-type fractions without annotation	[23]
CDSeq	Probabilistic model (LDA)	bulk RNA-seq Data	cell-type fractions	[24]
Linseed	Simplex topology	bulk RNA-seq Data	cell-type fractions without annotation	[25]
GS-NMF	Geometric structure guided nonnegative matrix factorization model	bulk RNA-seq Data	cell-type fractions without annotation	[26]

There are enrichment-based deconvolution methods. These methods estimate cell-type proportions in bulk RNA-seq data by leveraging predefined cell-type-specific marker genes rather than full reference expression profiles and rely on gene set enrichment analysis, rank-based statistics, and regression models to infer relative cell-type proportions. For example, Estimation of STromal and Immune cells in MAlignant Tumours using Expression data (ESTIMATE) [27], xCell [28], and BrainInABlender [29]. The enrichment based methods are beyond the scope of this review.

Both reference-based and reference-free methods have assumptions to achieve optimal performance. However, in practical applications, these assumptions may be violated due to technical and/or biological disparities between the reference and bulk tissue RNA-seq data. For example, different library preparation protocols and sequencing platforms between scRNA-seq and bulk tissue RNA-seq is a well-known technical factor; the inherent heterogeneity in gene expression among different tissues and cell types/stages could be a biological factor. In this study, our objective was to examine the robustness and resilience of the two different kinds of methods, by using four selected deconvolution algorithms, two reference-based, and two reference-free. Explicitly, CIBERSORTx, MuSiC, Linseed, and GS-NMF were chosen due to their relative popularity. We simulated *in silico* pseudo-bulk tissue RNA-seq datasets using four different tissue types, and three levels of cell composition variation. Pseudo-bulk tissues were also generated by shifting the distribution of the reference scRNA-seq data. The robustness and resilience of the four selected methods were evaluated in the deconvolution of these pseudo-bulk RNA-seq datasets.

## Study design

### Study goal

In this study, we focused on testing the robustness and resilience of two reference-based and two reference-free methods by evaluating their performance in various scenarios and identifying risk factors that impact the performance of these deconvolution methods. The two reference-based methods are CIBERSORTx [16] and MuSiC [19], while the reference-free methods are Linseed [25] and GS-NMF [26]. To test robustness, we used *in silico* pseudo-bulk RNA-seq data that mimic a wide range of scenarios and evaluated the performance of the methods. For resilience, we either intentionally altered the distribution of cell level gene expression profiles, or used two independent cell-level gene expression datasets to generate the pseudo-bulk data and reference data for deconvolution, respectively.

In essence, we simulated cell proportions of the pseudo-bulk tissues using a multivariate Dirichlet distribution with parameter $\gamma $. This parameter represents the expected proportion of cell types in bulk tissues. Subsequently, single cells were randomly sampled from an scRNA-seq dataset, and the chosen scRNA-seq profiles were directly summed up to create the pseudo-bulk tissue RNA-seq data. We then applied the four deconvolution methods to estimate the cell compositions in each pseudo-bulk tissue, respectively. The estimated cell proportions are compared with the ground truth using three metrics: Pearson’s correlation coefficient ($R$), the root mean square deviation ($RMSD$), and the mean absolute deviation ($MAD$).

### Notations and algorithms

For clarity in the subsequent sections, we introduce the mathematical notation here. The bulk tissue RNA-seq data are represented by a matrix $\mathbf{G}_{M\times n}$, where $M$ denotes the total number of genes and $n$ is the number of bulk tissue samples. Assuming that there are $K$ distinct cell types in the bulk tissue samples, we denote the expected expression levels of genes in these cells by a $M\times K$ matrix, $\mathbf{C}_{M\times K }$. The proportion of these cell types in bulk tissue samples is stored in a matrix $\mathbf{P}_{K\times n}$. The main objective of bulk tissue RNA-seq data deconvolution is to estimate the cell-type proportion, $\mathbf{P}$ as in Equation (1):


(1) \begin{align*}& \mathbf{G}=\mathbf{C}\mathbf{P}+\varepsilon.\end{align*}

Here, $\varepsilon $ is a random error term with $E(\varepsilon )=\mathbf{0}$. Reference-based methods utilize a reference dataset to estimate the expected cell-type-specific expression matrix $\mathbf{C}$, and assume that it is known in Equation (1) and only estimate matrix $\mathbf{P}$.

Specifically, CIBERSORTx is based on a $\nu $-support vector regression model [30], which was shown to be able to handle various challenges, including noise, unknown mixture content, and closely related cell types. Matrix $\mathbf{C}$ is a matrix of known gene expression profiles of various cell types. Each column of the matrix corresponds to a reference profile of a particular cell type, and each row corresponds to a gene. Signature genes are preselected by DE analysis of cell-level populations. With the known matrix $\mathbf{C}$, cell proportion matrix $\mathbf{P}$ can then be estimated using a $\nu $-support vector regression model. CIBERSORTx also incorporates batch effect correction when deconvolving data from multiple sources, and it is one of the most widely used deconvolution methods that have been applied to various types of bulk tissue, including but not limited to whole blood and solid tumor tissues. [16, 17]. MuSiC also estimates matrix $\mathbf{C}$, representing the average gene expression of each cell type, from a single-cell reference dataset. It utilizes a weighted nonnegative least squares (*w-nnls*) regression model to estimate the proportions of cell types [19]. The weights are calculated to reflect each gene’s reliability in distinguishing cell types, prioritizing those with low intra-cell-type variation and high inter-cell-type variation.

Reference-free deconvolution algorithms have been proposed particularly when there is a lack of a reference data set. Referring to equation (1), both matrices $\mathbf{C}$ and $\mathbf{P}$ are considered unknown and need to be estimated. Computational methods such as LINear Subspace identification for gene Expresion Deconvolution (Linseed) by Zaitsev *et al.* [25] and geometric structure-constrained nonnegative matrix factorization (GS-NMF) by Chen *et al.* [26] have been developed to estimate cell-type compositions. Ideally, the expression of cell-type-specific genes, the so-called marker genes, would be perfectly linearly correlated with the cell proportion of corresponding cell types. Reference-free methods take advantage of the mutual linearity to reveal the structure of the topological space of the bulk RNA-seq profiles. In particular, Linseed utilizes the mutual linearity to filter out non-informative genes and applies singular value decomposition to decide the number of cell types. It then uses the SISAL [31] algorithm construct the underlying simplex of known dimensionality, whose corners provide cell-type-specific genes and cell proportions. The GS-NMF was designed to improve the identifiability and uniqueness of the solution when deconvoluting the bulk RNA-seq data using the NMF algorithm. Traditional NMF estimates matrices $\mathbf{C}$ and $\mathbf{P}$ by solving the optimization problem: $(\hat{\mathbf{C}}, \hat{\mathbf{P}})=\text{argmin}_{\mathbf{C}\geq 0, \mathbf{P}\geq 0}||\mathbf{G-CP}||_{F}^{2}$. GS-NMF integrates the information from cell-type-specific marker genes as solvability constraints and the correlation graph as a manifold regularization, $(\hat{\mathbf{C}}, \hat{\mathbf{P}})=\text{argmin}_{\mathbf{C}\geq 0, \mathbf{P}\geq 0}||\mathbf{G-CP}||_{F}^{2}+\lambda _{1}\mathcal{F}_{1}(\mathbf{C})+\lambda _{2}\mathcal{F}_{2}(\mathbf{C})$ [26]. By incorporating two regularization terms within the NMF algorithm, GS-NMF enhances identifiability and improves the accuracy of cell proportion estimation.

### Simulation design

#### Cell-level datasets for pseudo-bulk tissue generation

**Figure 1:**
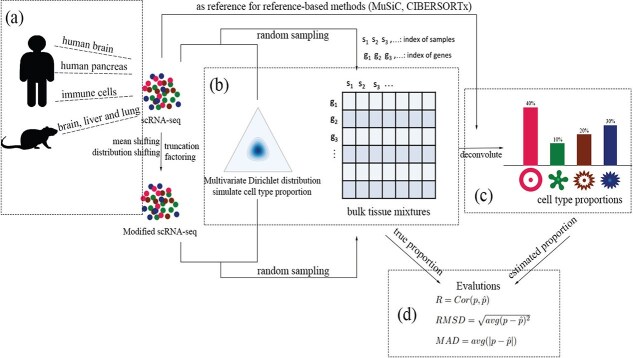
Study design. (a) A cell-level dataset is used to generated pseudo-bulk tissues. (b) Ideal mixing to generate pseudo-bulk tissue RNA-seq data. (c) Deconvolution for cell proportions. (d) Performance evaluation.

Four cell-level expression datasets of different tissue types are used for the generation of pseudo-bulk tissues in our simulations (Table 2). The first data set (GSE19830) contains 42 samples, including nine pure rat brain, liver, and lung cells and 33 mixtures of the three types of cells, in various mixing compositions [32]. Only nine pure cells are used in our simulations to generate pseudo-bulk tissue samples. The second data set includes 22 leukocyte subsets of mature human hematopoietic populations. These populations were derived from peripheral blood or culture conditions *in vitro* [17]. This data set has detailed cell-type annotation for each cell including subtypes, such as naive and memory B cells and CD8, CD4 naive, CD4 memory resting, CD4 memory activated, follicular helper, Tregs, and gamma delta T cells. In this study, we focus on six distinct cell types: B cells, T cells, NK cells, macrophages, dendritic cells, and mast cells by grouping all subtypes. The third data set (GSE81608) includes data on the scRNA sequence of human pancreatic islet cells that are from 12 nondiabetic and six T2D samples [33]. This data set has a total of 1492 cells, including pancreatic $\alpha $, $\beta $, $\delta $, and $\gamma $ cells. The last data set, GSE67835, comprises 466 single cells in healthy human brain samples [34]. This data set includes cells of nine distinct types, astrocytes, endothelial, replicating and quiescent fetal neurons, hybrid cells, microglia, regular neurons, oligodendrocytes, and oligodendrocyte precursor cells. We used five types: astrocytes, endothelial cells, microglia, neurons, and oligodendrocytes, i.e. a total of 267 cells, in our study to generate pseudo bulk brain tissue RNA-seq data. There are three additional data sets in Table 2: PBMC8K, E-MTAB-5061, and syn18485175. These datasets were either used as independent external reference data for deconvolution or to generate bulk tissue RNA-seq data. In particular, we used dataset PBMC8K to generate pseudo whole blood samples and used the LM22 as the reference dataset. The single nucleus RNA-seq data set (snRNA-seq) of the human brain [35] (syn18485175) was used as the reference data set when deconvoluting the pseudo human brain bulk tissues. And the T2D single-cell dataset [36] (E-MTAB-5061) as the reference dataset when deconvoluting pseudo T2D bulk tissue samples by SGE81608. In total, 12 ($4\times 3$) pseudo-bulk tissue RNA-seq data sets of four different tissue types and three levels of variation in cell composition were generated and used to assess the performance of the deconvolution methods (Fig. 1a).

**Table 2:** Cell level gene expression datasets

Data	GSE19830	LM22	GSE81608	GSE67835
Tissue type	rat brain, liver, lung	human blood	human pancreas	human brain
Platform	Affymetrix	Affymetrix	Illumina	Illumina
Num. of genes	13 841	11 845	39 849	22 088
Num. of cells	9	22	1492	267
Num. of cell types	3	6	4	5
Num. of subjects	NA	NA	18^*^	NA
Reference	[32]	[17]	[33]	[34]
**Data**	**PBMC8K**	**E-MTAB-5061**	syn18485175	
Tissue type	human peripheral blood	human pancreas	human brain	
Platform	Illumina	Smart-seq2	Illumina	
Num. of genes	32 738	25 453	17 926	
Num. of cells	8381	2209	75 060	
Num. of cell types	10	4	5	
Num. of subjects	NA	10^**^	48	
Reference	[39]	[36]	[35]	

$^{*}$ 12 healthy and six T2D samples.

$^{**}$ Six healthy and four T2D samples.

To perform a comprehensive resilience analysis, we systematically introduced controlled modifications to the generating datasets. We modified the cell-level data to generate pseudo bulk tissues and then estimated the cell proportions using the original, not modified data as the reference. We fixed the variation in cell composition at the medium level when testing resilience. Three distinct manipulative techniques were used:

(i) *Mean shifting*: augment expression level by adding 10%, 30%, 50%, and 70% of its average expression level in each cell type.
(ii) *Truncation*: selectively exclude cells from bulk tissue data generation by removing the top or bottom $10\%$ of expressed cells from each cell type. The remaining cells were then used to generate the bulk tissue data.
(iii) *Factoring*: a scaling factor was multiplied to the expression levels to allow both upward and downward adjustments. The scaling factors used were 0.4, 0.8, 1.2, and 1.8, respectively.

We also used a simulator, the scDesign simulator by Li *et al.* [37], to simulate scRNA-seq datasets. This simulator estimates the distribution parameters of the assumed probability model for gene expression levels based on real scRNA-seq data, enabling the generation of synthetic scRNA-seq data. In our experiments, we used scDesign to generate synthetic data for individual cells of human blood, brain, and pancreatic tissue. The synthetic cell-level datasets were then used to generate pseudo-bulk tissues, and the original single-cell dataset served as a reference for deconvolution.

Lastly, to assess the performance of the deconvolution methods in a more realistic setting, where the reference dataset and bulk tissue dataset can come from different labs/studies. Two independent cell-level datasets were used: one to generate pseudo-bulk samples and the other as a reference dataset. LM22 was used as a reference data when deconvoluting pseudo-bulk whole blood sample by using PBMC8K; single-cell dataset E-MTAB-5061 was used as reference when deconvoluting pseudo pancreatic samples generated by using GSE81608. And we used an snRNA-seq data set of the human brain [35] as the reference when deconvoluting the simulated pseudo bulk tissues. Although both scRNA-seq and snRNA-seq are potent techniques for studying gene expression at the cell level, they differ in the starting material and analyzed cell types. scRNA-seq captures intact cells and cytoplasmic RNA, while snRNA-seq isolates nuclei and captures nuclear RNA, making each technique suitable for distinct experimental contexts and sample types. Additionally, scRNA-seq results can vary depending on the platforms used. In this setting, our aim is to mimic the bulk tissue RNA-seq data deconvolution performed in real applications, where the reference dataset may come from different techniques or platforms.

#### Cell proportion generation

We simulate the proportion of cells in pseudo-bulk tissues using a multivariate Dirichlet distribution with parameter $\mathbf{p}=(p_{1}, p_{2}, \cdots , p_{K})$, where is a $K\times 1$ vector. $\gamma _{k}=p_{k}/\sum _{k=1}^{K}p_{k}$ is the expected proportion of the $k$th cell type in bulk tissue samples. The sum $p_{0}=\sum _{k=1}^{K}p_{k}$ controls the variation in cell compositions, a greater value of $p_{0}$ associates with a smaller variation. Our simulations consider three levels of variation—small, medium, and large—as detailed in Table 3. Specifically, for pseudo bulk generation using liver, lung, and brain cells, we used an equal mean proportion of $1/3$ for each cell type. For human blood pseudo-bulk, we used the expected proportions $\gamma _{blood}=(0.17, 0.43, 0.09, 0.14, 0.09, 0.09)$ for B cells, T cells. NK cells, Macrophages, dendritic cells, and mast cells, respectively. In pancreatic pseudo-bulk tissues, the mean proportions of $\alpha , \beta , \gamma , \delta $ cells were set to $\gamma _{islet}=(0.51, 0.33, 0.1, 0.05)$, respectively. Lastly, for human brain pseudo-bulk tissues, the mean proportions were $\gamma _{brain}=(0.23, 0.07, 0.06, 0.49, 0.14)$ for astrocytes, endothelial cells, microglia, neurons, and oligodendrocytes cells, respectively. These proportions were chosen to reflect real compositions of cells in human tissues. R package, *LaplacesDemon*, was used to generate random samples from the multivariate Dirichlet distribution.

**Table 3:** Values of parameter $\mathbf{p}$ for the Multivariate Dirichlet distribution used for simulations

Data set	Small variation	Medium variation	Large variation
GSE19830	(3.3, 3.4, 3.3)	(3.3, 3.4, 3.3)/10	(3.3, 3.4, 3.3)/100
LM22	(1, 2.5, 0.5, 0.8, 0.5, 0.5)	(1, 2.5, 0.5, 0.8, 0.5, 0.5)/10	(1, 2.5, 0.5, 0.8, 0.5, 0.5)/100
GSE81608	(5.4, 3.5, 1.1, 0.5)	(5.4, 3.5, 1.1, 0.5)/10	(5.4, 3.5, 1.1, 0.5)/100
GSE67835	(6.2, 2, 1.6, 13.1, 3.8)	(6.2, 2, 1.6, 13.1, 3.8)/10	(6.2, 2, 1.6, 13.1, 3.8)/100

#### Ideal mixing for pseudo-bulk tissue RNA-seq data

After simulating the proportions of cells, we randomly sampled single cells from the corresponding scRNA-seq dataset with replacement. The read counts/expression levels of the selected single-cell RNA profiles were directly added to be the total expression of the genes for the bulk tissue samples (Fig. 1b). We refer to this method as the ”ideal mixing approach.” A total of 50 mixture samples were generated for each dataset, except for the T2D dataset, where 100 samples were generated—50 for the healthy group and 50 for the T2D group.

For all simulated bulk tissue samples, we discarded genes with fewer than 10 read counts in at least $80\%$ of the samples and applied column normalization. For the human brain tissues, we used the top $1000$ marker genes by McKenzie *et al*. [38] for each of the five brain cell types considered in this study, resulting a total of 5000 genes included in our analyses for human brain tissues.

### Evaluation metrics

The performance of these methods are compared based on three quantitative metrics: (a) Pearson’s correlation correlation, $R= Cor(p,\hat{p})$, (b) Root mean squared deviation, $RMSD=\sqrt{\sum _{k=1}^{K}\frac{(p_{k}-\hat{p}_{k})^{2}}{K}}$, and (c) Mean Absolute Deviation, $MAD = \frac{1}{K}\sum _{k=1}^{K}|p_{k}-\hat{p}_{k}|$, where $p$ and $\hat{p}$ represent the ground truth and the estimated proportions of the cells, respectively (Fig. 1d).

## Results

Through intensive simulations, we gained valuable insights into the performance of these deconvolution methods in terms of robustness and resilience.

### Robustness

Overall, the two reference-based methods, CIBERSORTx and MuSiC, consistently outperform the two reference-free methods, Linseed and GS-NMF, across various tissue types and levels of cell composition variation, particularly when the reference dataset used is the dataset used to generate the pseudo-bulk tissues.

**Figure 2:**
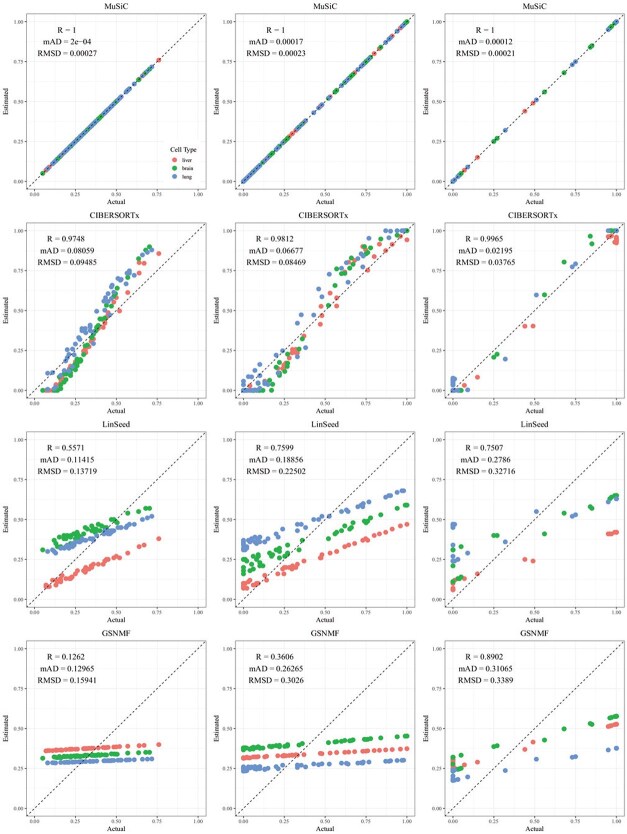
Comparison of estimated versus actual cell proportions in pseudo-bulk samples from dataset GSE19830. The columns (left to right) represent small, medium, and large cellular composition variations. The rows (top to bottom) show results from MuSiC, CIBERSORTx, LinSeed, and GS-NMF. The colors indicate liver (red), brain (green), and lung (blue) proportions.

**Figure 3:**
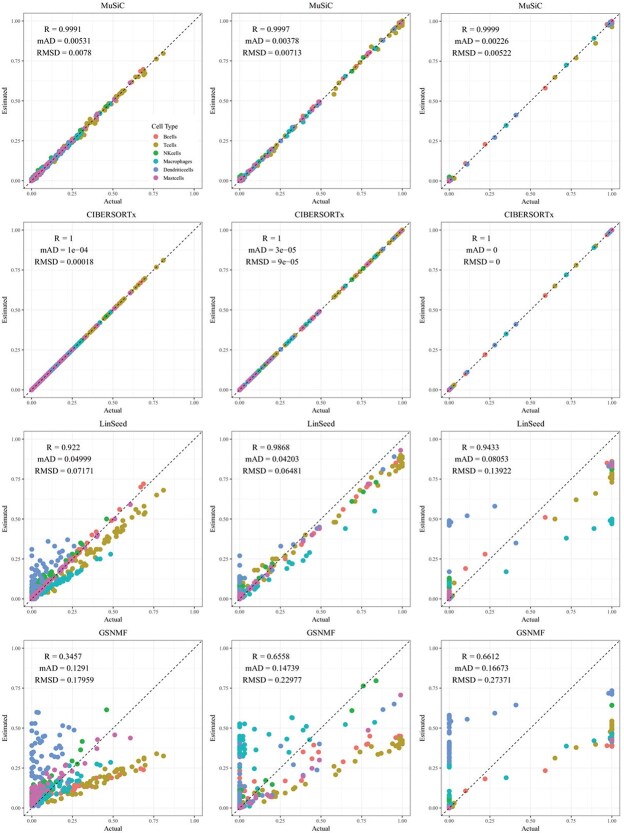
Comparison of estimated versus actual cell proportions in pseudo-bulk samples from dataset LM22. The columns (left to right) represent small, medium, and large cellular composition variations. The rows (top to bottom) show results from MuSiC, CIBERSORTx, LinSeed, and GS-NMF. The colors indicate B cells (red), T cells (gold), NK cells (green), Macrophages (cyan), Dendritic cells (blue), and Mast cells (magenta).

**Figure 4:**
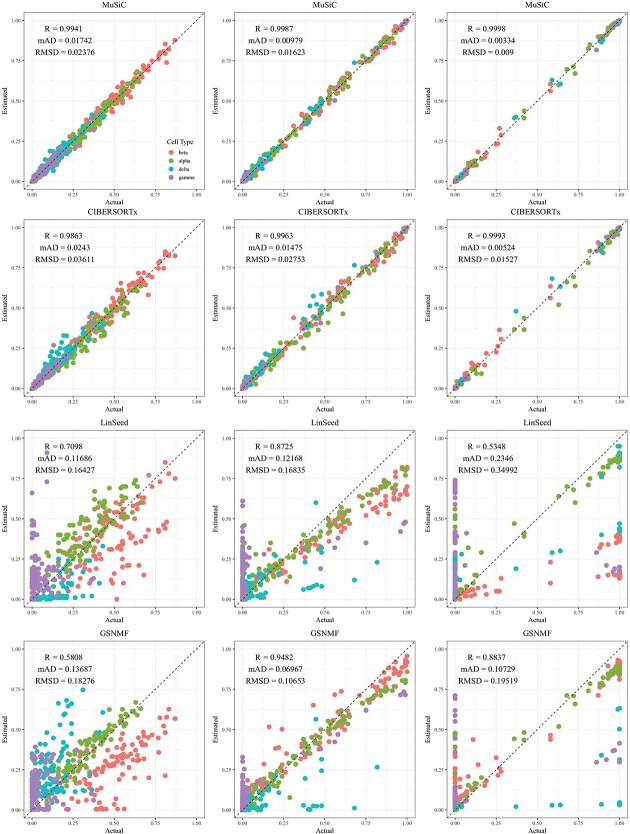
Comparison of estimated versus actual cell proportions in pseudo-bulk samples from dataset GSE81608. The columns (left to right) represent small, medium, and large cellular composition variation. The rows (top to bottom) show results from MuSiC, CIBERSORTx, LinSeed, and GS-NMF. The colors indicate beta (red), alpha (green), delta (cyan), and gamma (purple).

**Figure 5:**
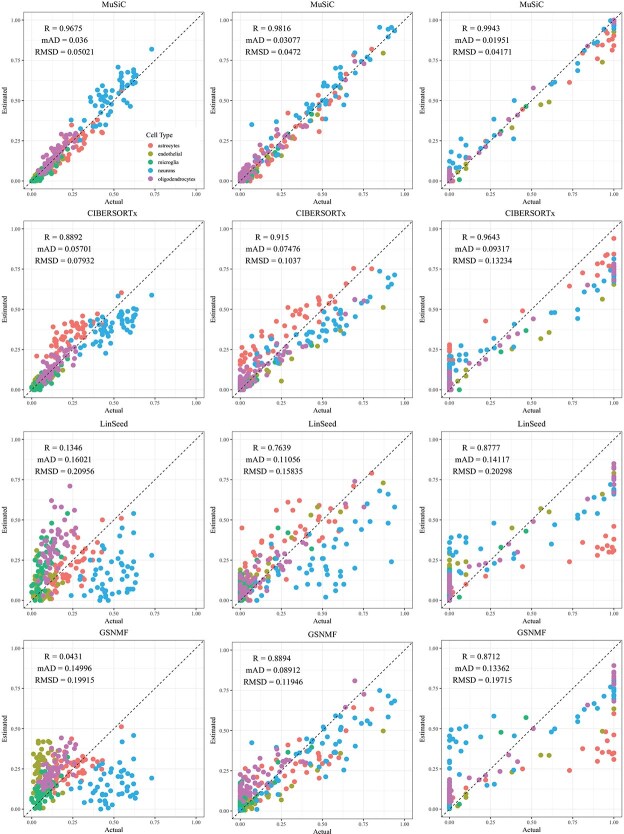
Comparison of estimated versus actual cell proportions in pseudo-bulk samples from dataset GSE67835. The columns (left to right) represent small, medium, and large cellular composition variation. The rows (top to bottom) show results from MuSiC, CIBERSORTx, LinSeed, and GS-NMF. The colors indicate astrocytes (red), endothelial (gold), microglia (green), neurons (blue), and oligodendrocytes (magenta).

CIBERSORTx and MuSiC demonstrated comparable performance at different levels of variation in cellular composition, with Pearson’s correlation coefficients between the estimated and true cell proportions being close to 1, as shown in Figs 2–5. When comparing their performance across different bulk tissue types, estimates for pancreatic and brain tissues are slightly less accurate than those for liver, lung, brain pseudo-bulk tissues, and whole blood pseudo-bulk samples. For example, the observed RMSD for MuSiC is 0.00023, 0.00713, 0.0162, and 0.0472 for the liver, lung, brain, whole blood, pancreatic, and brain tissue samples, respectively, under medium variation in cell proportions. The corresponding RMSD for CIBERSORTx are 0.0847, 0.00009, 0.0275, and 0.1037, respectively. Similar patterns were observed in the Pearson’s correlation coefficient and MAD (Figs 2–5).

Linseed struggles to accurately estimate cell proportions when there is minimal variation in cellular composition. But, its performance improves significantly as the variation increases medium level, and then decreases slightly when the variation level gets to large. For example, in pseudo-bulk tissues for the liver, lung, and brain, Pearson’s correlation coefficients between the estimated cell proportions and the ground truth for Linseed are 0.557, 0.760, and 0.751, respectively, for the small, medium, and large variation levels (third row in Fig. 2). Similar pattern was observed in pseudo whole blood and pancreatic tissues. However, for pseudo human brain tissues, the Pearson’s correlation coefficients for the estimates keep increasing as the variation level increases. GS-NMF faces the same challenge. Its Pearson’s correlation coefficients are 0.126, 0.361, and 0.890 for pseudo liver, brain, and lung mixtures (fourth row in Fig. 2); and 0.346, 0.656, and 0.661 for pseudo whole blood samples (fourth row in Fig. 3). For pseudo pancreatic and brain tissues, GS-NMF also achieved the best performance at medium level of variation, with Pearson’s correlation coefficients at 0.581, 0,948, and 0.884 for pseudo pancreatic samples (Fig. 5), and 0.043, 0.889, and 0.871 for pseudo brain tissues, respectively (Fig. 5). Compared with GS-NMF, Linseed provides better estimates when the variation in cell proportions is small to medium, especially for brain, liver, lung mixtures, and pseudo whole blood mixtures. However, as the variation in cell proportions increases, GS-NMF can outperform Linseed. For example, in pseudo pancreatic tissues, GS-NMF achieves Pearson’s correlation coefficients of 0.948 and 0.884 at medium and large variation levels, compared with Linseed’s results of 0.873 and 0.535, respectively (Fig. 4). In pseudo brain tissues, GS-NMF demonstrates performance comparable with Linseed (Fig. 5).

In summary, when pseudo-bulk tissues are generated by ideal mixing, as in our simulations, and the same cell-level dataset is used as a reference, the reference-based methods exhibit robust performance in estimating cell proportions across various tissue types and levels of cellular composition variation. Two major sources of variation in the simulated bulk tissue samples were observed to influence the performance of these methods: variation in cell-level gene expression profiles and variation in cell compositions.

**Figure 6:**
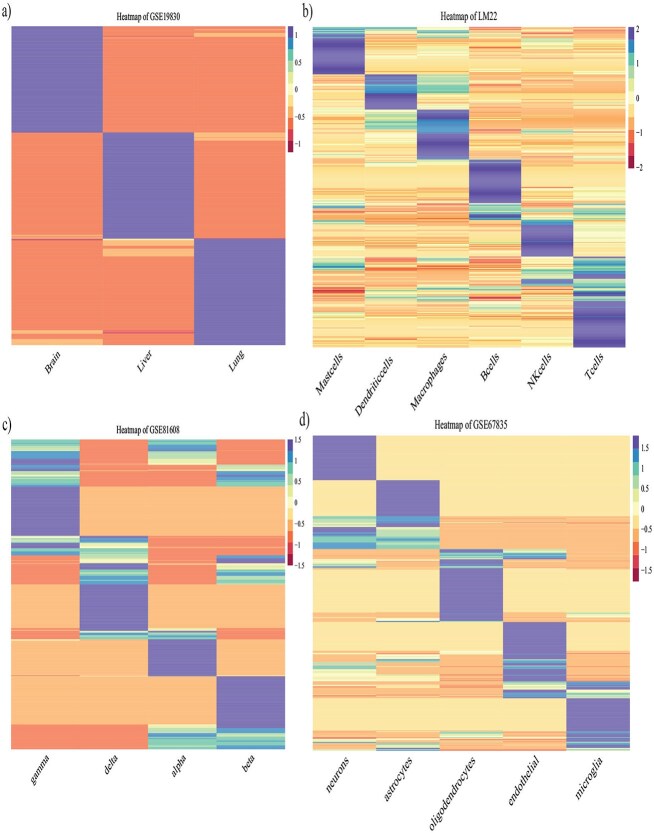
Heatmap of correlation between signature genes selected by CIBERSORTx: (a) GSE19830, (b) LM22, (c) GSE81608, and (d) GSE67835.

**Variation in cell-level gene expression profiles.** The four scRNA-seq datasets used varied in the number of cells and tissue types. The liver, lung, and brain dataset contains only nine single cells and shows the most distinct gene expression patterns in the three cell types, as illustrated in Fig. 6(a). The signature genes selected by CIBERSORTx display very different expression patterns in liver, lung, and brain cells. Additionally, due to the limited number of cells, random sampling could result in the same cell being selected multiple times, leading to a smaller sampling variation compared with the other data sets. The LM22 data set exhibits similar characteristics. Although LM22 contains more cells, the data set has been carefully preprocessed and thoroughly cleaned, resulting in a much clearer cell-type-specific gene expression pattern, as shown in the heatmap (Fig. 6b). In contrast, the T2D and brain cell datasets show more variable gene expression patterns: less unique expression pattern in marker genes, greater variability within cell types, and a larger number of cells, contributing to greater overall variation in gene expression at the cell level. In general, these differences in the results highlight how the number of cells, tissue types, and biological techniques used for the cell-level gene expression measurement can significantly affect the variation in the cell-level data, and therefore influence the clarity and consistency of the deconvolution.

**Variation in cell proportions.** In these pseudo-bulk tissues, created through ideal mixing, the performance of reference-based methods remains largely unaffected. These methods consistently outperform others in terms of Pearson’s correlation coefficient, RMSD, and MAD. On the other hand, the degree of variation in cellular composition significantly affects the performance of reference-free methods. Greater variation correlates with more accurate estimations. The reasons for this association are probably two-fold: (1) variation in cell proportions helps identify marker genes. Reference-free methods assume a linear relationship between the cell proportion and the corresponding marker gene expression levels. When cell proportions vary widely, the linearity in expression levels becomes more pronounced, making marker genes easier to identify. (2) Mathematically, the identifiability condition is better satisfied when deconvolution takes advantage of correctly identified marker genes, leading to more precise results. In summary, a greater variation in cell composition enhances the accuracy of reference-free methods, while reference-based methods are affected at a minimum level and consistently perform well regardless of this variation.

### Resilience

We also evaluated the performance of these methods in conditions where there are discrepancies between the reference data and the true profiles of individual cells in bulk tissue samples. We examined three levels of discrepancy: (1) artificially manipulated scRNA-seq data for bulk tissue generation; (2) used synthetic scRNA-seq data by a simulator for bulk tissue generation; (3) used independent real cell-level data sets for pseudo-bulk tissue generation and reference, respectively. The following section summarizes our findings on the resilience of these methods in handling such discrepancies.

**Artificial manipulation by mean shifting, truncation, and factoring.** Our analyses results indicate that mean shifting and scaling have a minimal impact on the performance of the four selected deconvolution methods. MuSiC, CIBERSORTx, and GS-NMF maintained Pearson’s correlation coefficients similar to those observed in the non-manipulated scenario across tissue types and cellular composition variation levels. However, Linseed showed some variability in the estimation of cell proportions in pseudo-bulk pancreatic tissues, with Pearson’s correlation coefficients of 0.440, 0.803, 0.648, and 0.742 when 10%, 30%, 50%, and 70% of mean values were added, respectively. This contrasts with a coefficient of 0.8725 in the non-manipulated case (Supplementary Figure 3). Linseed and GS-NMF both exhibited improved performance when deconvoluting pseudo-bulk brain tissues with a higher percentage of cell-type-specific mean expression values added. Our designed factoring experiments do not show any significant changes in the performance of the four selected deconvolution methods (Supplementary Figures 5–8).

Removing the top or bottom $10\%$ of cells according to their overall expression level barely affects the performance of the reference-based methods, with Pearson’s correlation coefficients remaining almost 1. Interestingly, truncating the top or bottom cells improves the performance of GS-NMF, which has RMSD values of 0.303, 0.230, 0.107, and 0.119 for the four different types of original pseudo bulk tissues, and 0.301, 0.068, 0.103, and 0.106 when the top $10\%$ highly expressed cells were removed, and 0.301, 0.205, 0.101, and 0.108 when the bottom $10\%$ of cells were removed (bottom panel, Supplementary Figures 9–12). This observation indicates that the GS-NMF method might be sensitive to abnormal cells, especially extremely highly expressed cells. The results for Linseed are different; it has improved performance in pseudo whole blood and brain tissues, while worsened performance in pseudo pancreatic tissues, and about the same results in pseudo liver, lung, and brain mixtures. This observation suggests the performance of Linseed may be tissue-dependent.

**Figure 7:**
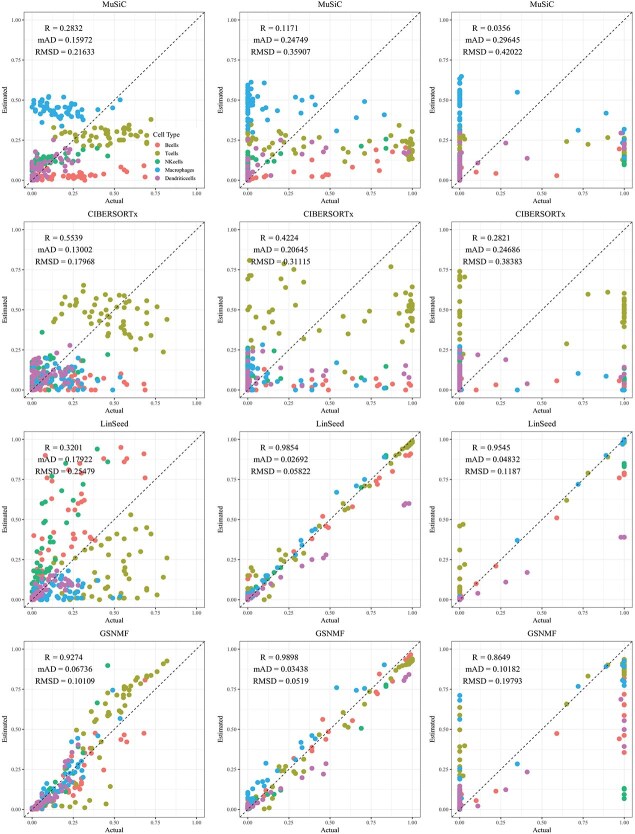
Estimated versus true cell proportions for dataset generated by the scDesign simulator using PBMC8K. The columns (left to right) represent small, medium, and large variation in cellular composition. The rows (top to bottom) correspond to deconvolution method: MuSiC, CIBERSORTx, Linseed, and GS-NMF. The colors represent different cell types: B cells (red), T cells (gold), NK cells (green), Macrophages (cyan), Dendritic cells (blue), and Mast cells (magenta).

**Figure 8:**
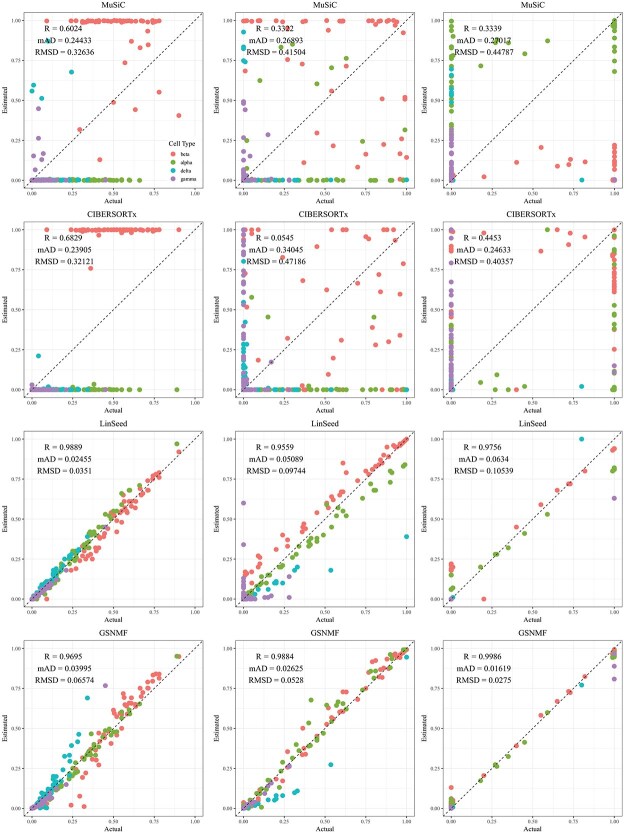
Estimated versus true cell proportions for dataset generated by scDesign simulator using GSE81608. The columns (left to right) represent small, medium, and large variation in cellular composition. The rows (top to bottom) correspond to deconvolution method: MuSiC, CIBERSORTx, Linseed, and GS-NMF. The colors represent different cell types: beta (red), alpha (green), delta (cyan), and gamma (purple).

**Figure 9:**
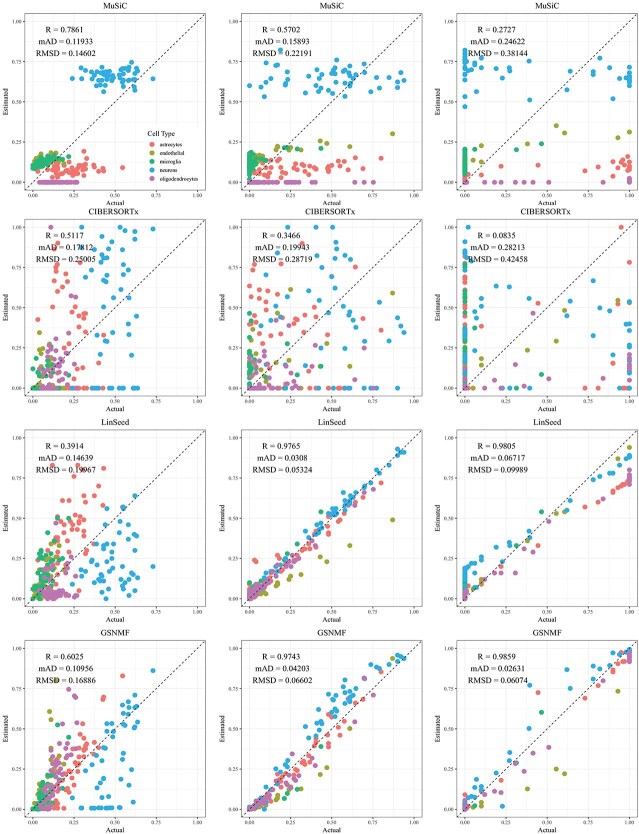
Estimated versus true cell proportions for dataset generated by the scDesign simulator using GSE67835. The columns (left to right) represent small, medium, and large variation in cellular composition. The rows (top to bottom) correspond to deconvolution method: MuSiC, CIBERSORTx, Linseed, and GS-NMF. The colors represent different cell types: astrocytes (red), endothelial (gold), microglia (green), neurons (blue), and oligodendrocytes (magenta).

When using artificially simulated scRNA-seq to generate pseudo bulk tissue data and the real scRNA-seq dataset as reference, the performance of reference-based methods is greatly affected (Figs 7–9). Especially for CIBERSORTx, Pearson’s correlation coefficients in the three kinds of simulated bulk tissue samples are dramatically smaller than when the same scRNA-seq data set for bulk tissue generation were used as reference. Not surprisingly, the performance of the two reference-free methods improves across all levels of variation in cellular composition. The Pearson’s correlation coefficient for GS-NMF are 0.923, 0.990, and 0.865 for pseudo-bulk blood samples: 0.6025, 0.9743, and 0.986 for pseudo brain tissue samples; and 0.970, 0.999, and 0.988 for pseudo pancreatic tissues at the three levels of variation in cellular composition, respectively. Similar patterns in RMSD and MAD can be seen in these figures. This improvement is mainly because the synthetic scRNA-seq data by the simulator are much less variable than the original real data, as they were generated by an assumed probability distribution. For the reference-based deconvolution methods, however, the performance is much worse presumably because of the discrepancy. The obtained Pearson’s coefficients by CIBERSORTx are much smaller than that of the other methods, and the RMSD and MAD are much bigger. This is one scenario where the fundamental assumption of regression-based methods—that cell-type-specific expression levels are known and observed with negligible errors—is violated. We see a decrease in the performance for both reference-based methods based on the three evaluation metrics in this simulation scenario. Reference-free methods have better resilience because they do not rely on external reference data.

**Figure 10:**
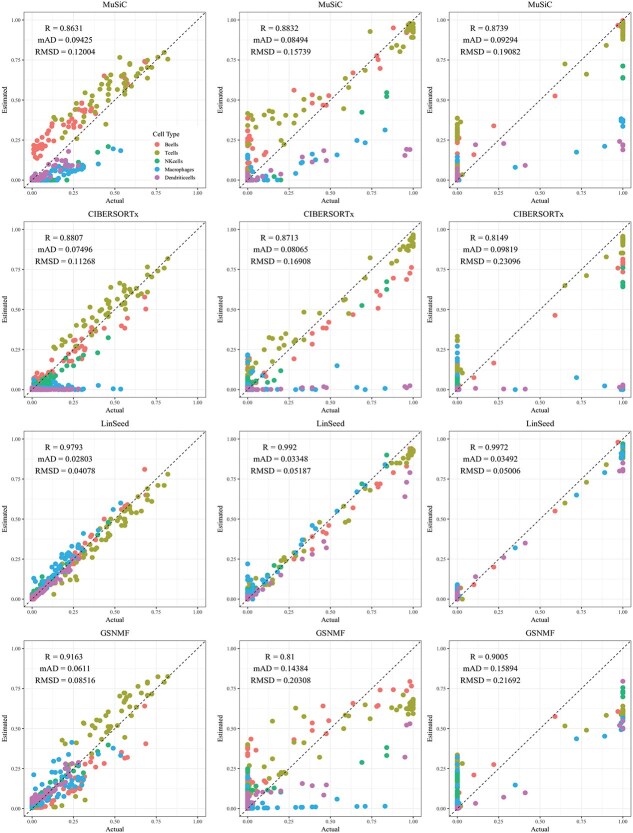
Resilience results: estimated versus true cell proportions for pseudo-bulk whole blood samples generated by using PBMC8K and analyzed by using LM22 as reference for reference-based methods. The columns (left to right) represent small, medium, and large variation in cellular composition. The rows (top to bottom) correspond to deconvolution method: MuSiC, CIBERSORTx, Linseed, and GS-NMF. The colors represent different cell types: B cells (red), T cells (gold), NK cells (green), Macrophages (blue), Dendritic cells (magenta).

**Figure 11:**
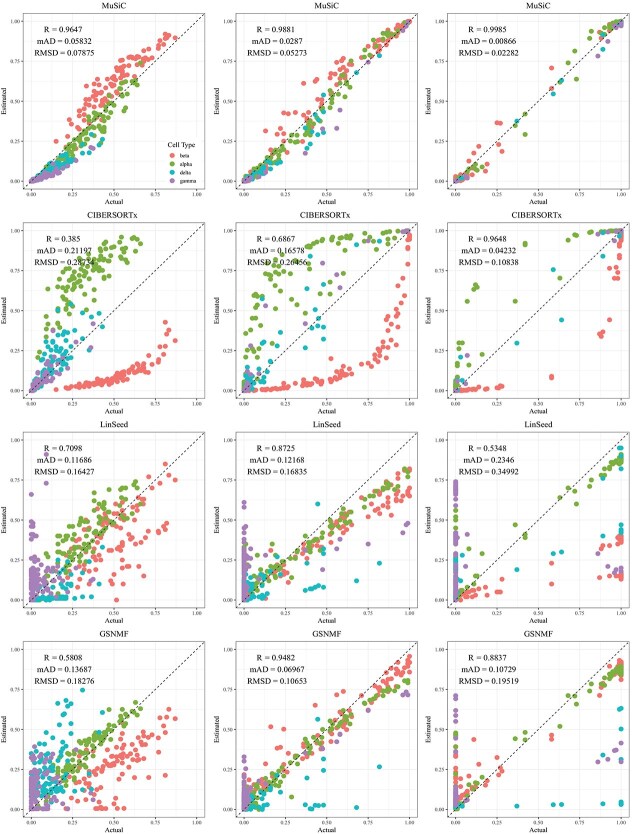
Resilience results: estimated versus actual cell proportions for pseudo-bulk tissues generated by GSE81608 dataset and E-MTAB-5061 dataset was used as reference for deconvolution. The columns (left to right) represent small, medium, and large variation in cellular composition. The rows (top to bottom) correspond to deconvolution method: MuSiC, CIBERSORTx, LinSeed, and GS-NMF. The colors represent distinct cell types: beta (red), alpha (green), delta (cyan), and gamma (purple).

**Figure 12:**
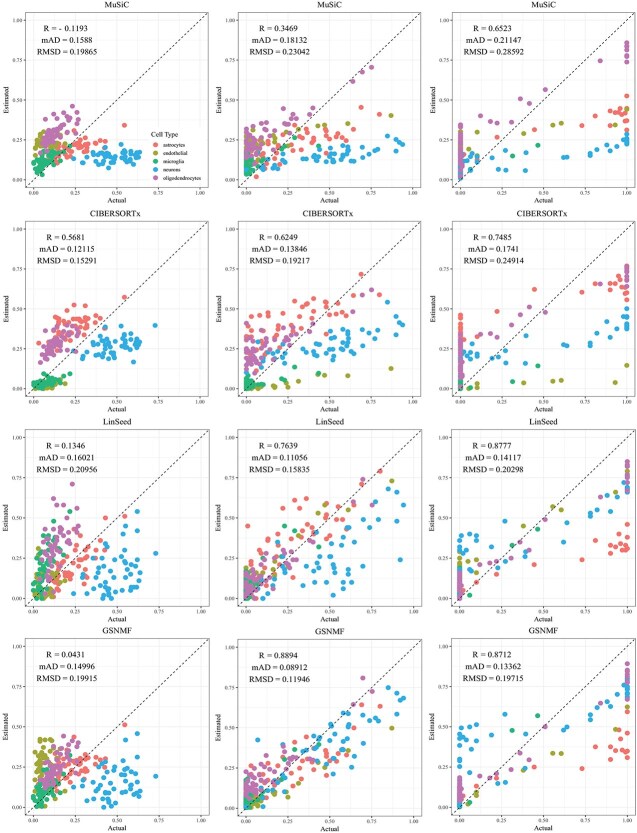
Resilience results: estimated versus actual cell-type proportions for pseudo-bulk tissues generated by GSE67835 dataset and dataset syn18485175 was used as reference for deconvolution. The columns (left to right) represent small, medium, and large variation in cellular composition. The rows (top to bottom) correspond to a different deconvolution method: MuSiC, CIBERSORTx, LinSeed, and GS-NMF. The colors represent distinct cell types: astrocytes (red), endothelial cells (gold), microglia (green), neurons (blue), and oligodendrocytes (magenta).

To further validate this finding in realistic settings, we conducted three more analyses using a real external, independent reference data set for deconvolution. The two reference-based methods show reduced estimation accuracy, while Linseed and GS-NMF maintain similar performance because no external reference data are needed. For pseudo whole blood samples, MuSiC achieved cell proportion estimates with Pearson’s correlation coefficients of 0.863, 0.883, and 0.874 across the three levels of cellular composition variation. CIBERSORTx obtained coefficients of 0.881, 0.871, and 0.815, respectively. LinSeed demonstrated higher correlations, with coefficients of 0.979, 0.992, and 0.997, while GS-NMF achieved 0.916, 0.81, and 0.901, respectively (Fig. 10). In bulk pancreatic tissues, MuSiC performs consistently well across the variation levels of cell composition we considered, with Pearson’s correlation coefficients of 0.965, 0.988, and 0.999, respectively. However, CIBERSORTx showed slightly worse results compared with ideally mixed pseudo-bulk tissues that we discussed previously, with Pearson’s correlation coefficients of 0.385, 0.687, and 0.965, respectively. Not surprisingly, the performance of reference-free methods is not affected as they do not rely on external reference data for estimation (Fig. 11). Furthermore, when pseudo-brain tissue samples were decomposed using external snRNA sequencing data, the performance of both reference-based methods dropped. Pearson correlation coefficients for MuSiC were −0.120, 0.347, and 0.652, while GS-NMF has 0.043, 0.889, and 0.871. Additionally, Pearson’s correlation coefficients for CIBERSORTx are 0.568, 0.625, and 0.749, whereas Linseed obtained 0.135, 0.764, and 0.878, respectively (Fig. 12). Similar trends can be seen in the bar plots of RMSD and MAD as illustrated in (Figs 10–12). These results suggest that reference-free methods could achieve better estimates than reference-based methods, especially when no reliable reference dataset is available and particularly when cell composition variability reaches at least a medium level.

## Discussion

In this study, we utilized real cell-level gene expression datasets to generate *in silico* pseudo-bulk RNA-seq data and evaluated four computational deconvolution methods: CIBERSORTx, MuSiC, Linseed, and GS-NMF. These methods were assessed based on their ability to estimate cell proportions using Pearson’s correlation coefficient, RMSD, and MAD as evaluation metrics. The primary objective was to evaluate the robustness in various tissue types and levels of variation in cell composition and resilience of these methods when there was a discrepancy between the reference data set and the real transcriptome of cells in bulk tissues.

In our simulation studies, we used cell-level datasets from four different tissue types and generated bulk tissue samples with three levels of variation in cellular composition to examine the robustness of the selected computational methods. For discrepancy between reference data and the real cell-level transcriptome in bulk tissues, we used manipulated cell-level data, simulator-generated synthetic scRNA-seq data, and external independent scRNA-seq datasets to generate pseudo-bulk tissues.

Both reference-based methods (CIBERSORTx and MuSiC) demonstrated strong robustness across tissue types and levels of variation in cell composition. All three evaluation metrics consistently showed better performance from these methods, particularly when the pseudo-bulk tissues were generated by ideal mixing, and the true cell-level gene expression profiles were used as the reference. CIBERSORTx and MuSiC provided much more accurate estimates, with higher correlation with the true cell proportions and lower RMSD and MAD values. The two methods performed comparable in our simulations.

On the other hand, the reference-free methods (Linseed and GS-NMF) showed variable results depending on the level of variation in cellular composition and tissue type. Linseed performed the best in pseudo-whole-blood tissues, with Pearson’s correlation coefficients of 0.922, 0.986, and 0.943 at the levels of small, medium and large variation, respectively. However, its performance in other tissue types, especially at low level of variation, was less satisfactory. GS-NMF performed the best when the level of variation in cellular composition is large. The differences in performance between the two reference-free methods were largely due to their ability to identify reliable marker genes. In general, the variation in cell composition proved beneficial for the identification of marker genes. This trend was consistent with the RMSD and MAD metrics.

When there was a discrepancy between the reference dataset and the single cells in bulk tissue, the performance of reference-based methods was significantly affected, as the fundamental assumption of the regression models was violated. In contrast, reference-free methods, which learn the underlying structure of bulk tissues without relying on external reference data, showed resilience to this discrepancy. However, some variation in cell proportions was necessary for their optimal performance. GS-NMF, as a more mathematical approach, did not account for the variability in scRNA-seq data when selecting marker genes, whereas Linseed incorporated a permutation procedure, resulting in smaller errors. Similarly, CIBERSORTx, which employs a support vector regression model, performed better in noisier datasets, while MuSiC, with its regression model, performed better with less variable data. These findings were further validated in our final simulation, where pseudo-bulk tissue data were generated using one scRNA-seq dataset, while an external independent scRNA-seq dataset was used as the reference.

In summary, we thoroughly investigated the performance of two reference-based and two reference-free methods for the deconvolution of bulk tissue RNA-seq data. Various scenarios were considered, including different tissue types, cell composition variation levels, and discrepancies between reference datasets and single-cell transcriptomes. Overall, reference-based methods outperformed reference-free methods, particularly when pseudo-bulk tissues were generated using the same single-cell profiles used for deconvolution. However, when discrepancies between datasets were introduced, reference-free methods sometimes outperformed their reference-based counterparts. These insights can serve as general guidelines for selecting a deconvolution method in real-world applications. We also observed that variation in scRNA-seq and cell composition are critical factors in improving and developing more efficient computational deconvolution methods.

Nonetheless, our study has limitations. While we considered a wide range of scenarios, our simulations were not exhaustive. Tissue types extend beyond the four we investigated, and numerous deconvolution algorithms exist beyond the ones we examined. Additionally, we relied on three commonly used evaluation metrics without critically assessing their suitability for this analysis. Although we introduced significant variation in cell composition to study its impact on deconvolution, the extent of this variation is typically unrealistic in real bulk tissue samples. However, exploring how such variation can be leveraged for algorithm development remains a valuable direction for future research.

**Key Points**

- Understanding the cellular composition of bulk tissue samples, which comprise a mixture of different cell types, is critical for elucidating the true causes of gene expression variation within these samples.
- Many computational algorithms were developed to deconvolute bulk tissue RNA-seq data for cell compositions, including reference-based methods that utilize external cell-level reference data; and reference-free methods that do not require external reference data. Each has distinct advantages and limitations.
- This study examines the **robustness** and **resilience** of four selected deconvolution algorithms and identifies risk factors influencing their performance through intensive simulations using *in silico* pseudo-bulk tissue data.
- This study provides significant insights into the factors affecting bulk tissue deconvolution performance, which are essential for guiding users and advancing the development of more powerful and reliable algorithms in the future.

## Supplementary Material

supplementary_Figures_bbaf264

supplementary_Figures_bbaf264

## Data Availability

The simulated pseudo-bulk RNA-seq datasets and python code for GS-NMF algorithm are available at our GitHub page: https://github.com/sxu509/benchmarkingstudy.
