# Supplementary material for: Robustness and resilience of computational deconvolution methods for bulk RNA sequencing data
Source: Brief Bioinform. 2025 Jun 12;26(3):bbaf264. doi: 10.1093/bib/bbaf264 (PMC12159287; doi:10.1093/bib/bbaf264)
Supplement: supplementary_Figures_bbaf264 [file supplementary_figures_bbaf264.pdf]

1 Supplementary Figures-Robustness

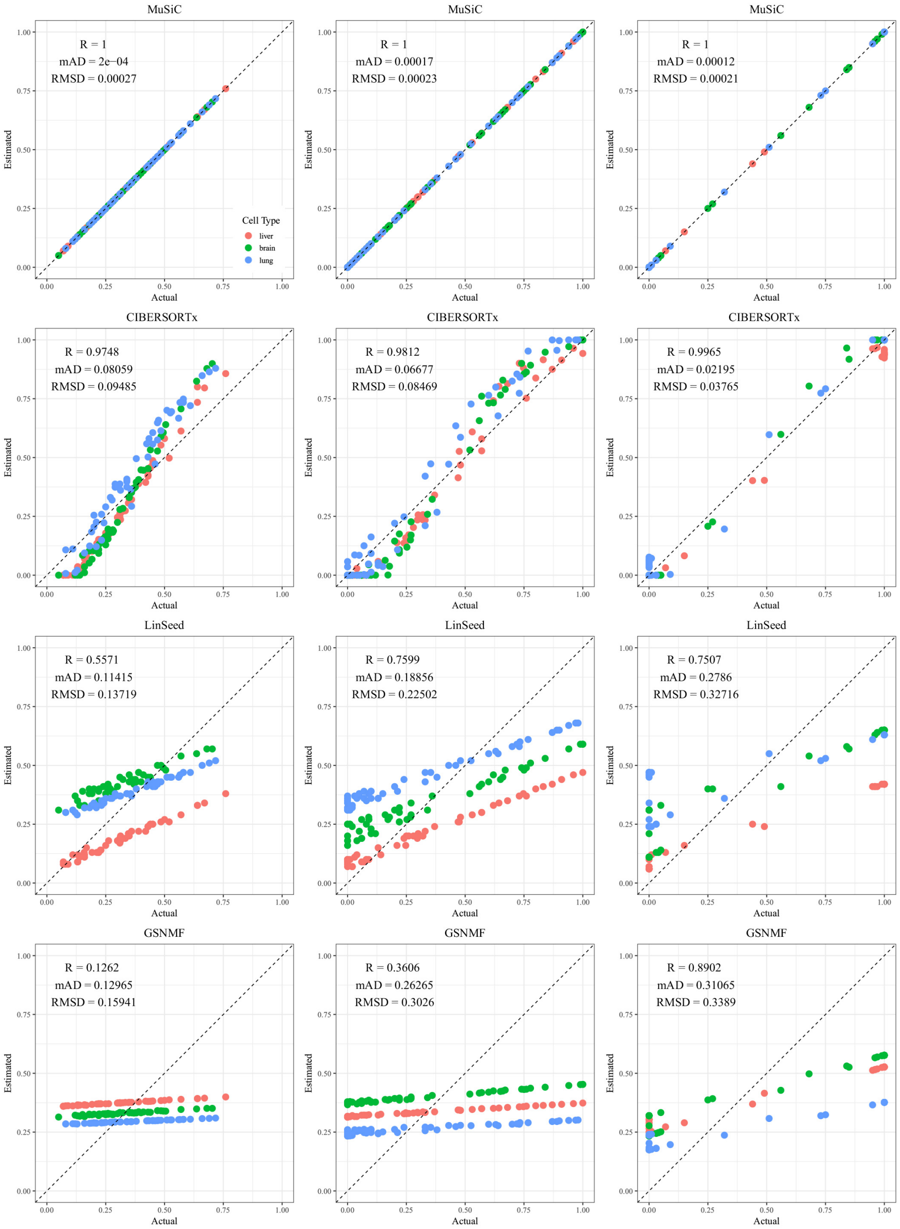

Figure 1: Comparison of estimated vs. actual cell proportions in pseudo-bulk samples from dataset GSE19830. Columns (left to right) represent small, medium, and large cellular composition variations. Rows (top to bottom) show results from MuSiC, CIBERSORTx, LinSeed, and GS-NMF. Colors indicate liver (red), brain (green), and lung (blue) proportions.

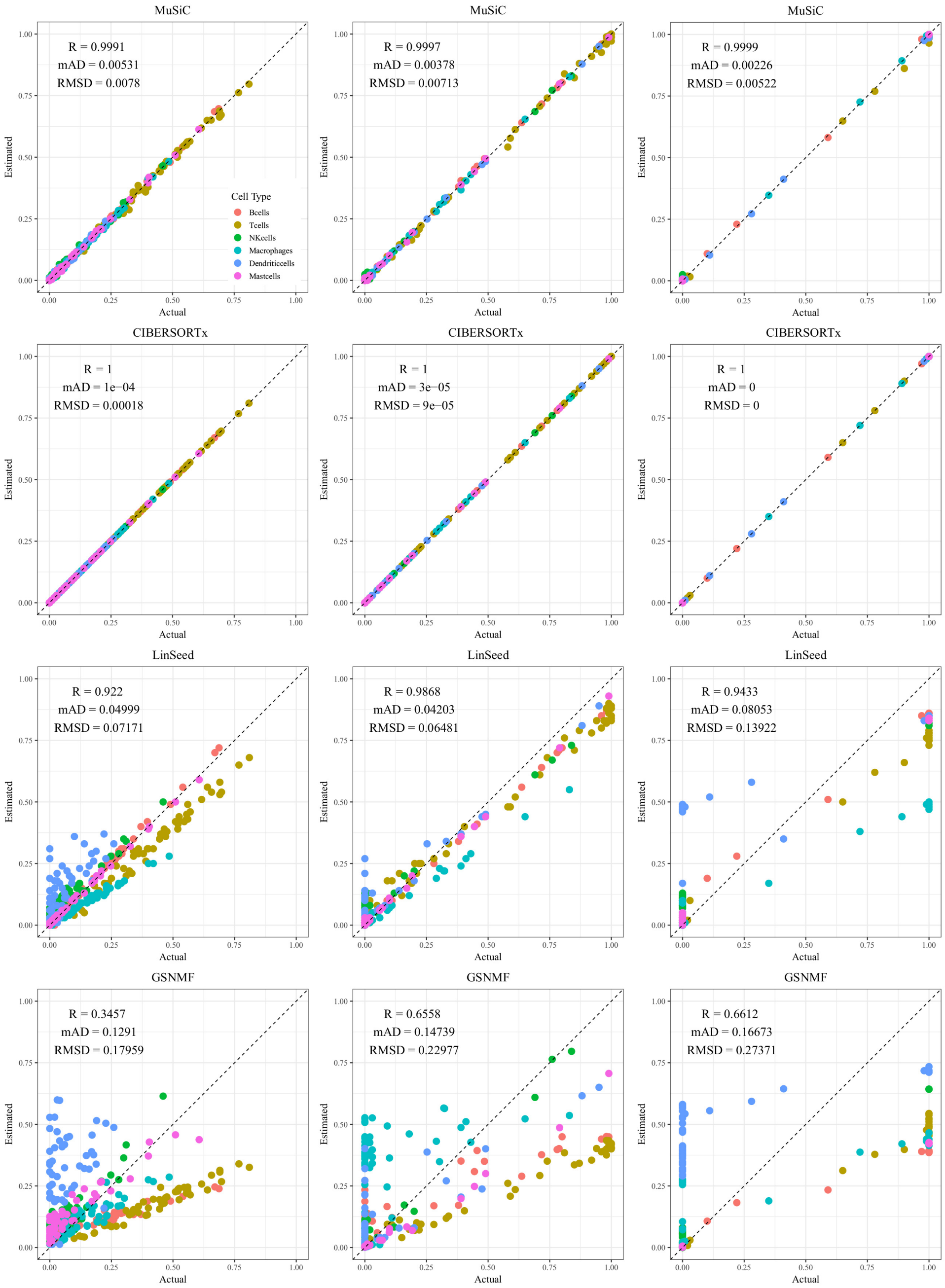

Figure 2: Comparison of estimated vs. actual cell proportions in pseudo-bulk samples from dataset LM22. Columns (left to right) represent small, medium, and large cellular composition variations. Rows (top to bottom) shows results from: MuSiC, CIBERSORTx, LinSeed, and GS-NMF. Colors indicate B cells (red), T cells (gold), NK cells (green), Macrophages (cyan), Dendritic cells (blue), and Mast cells (magenta).

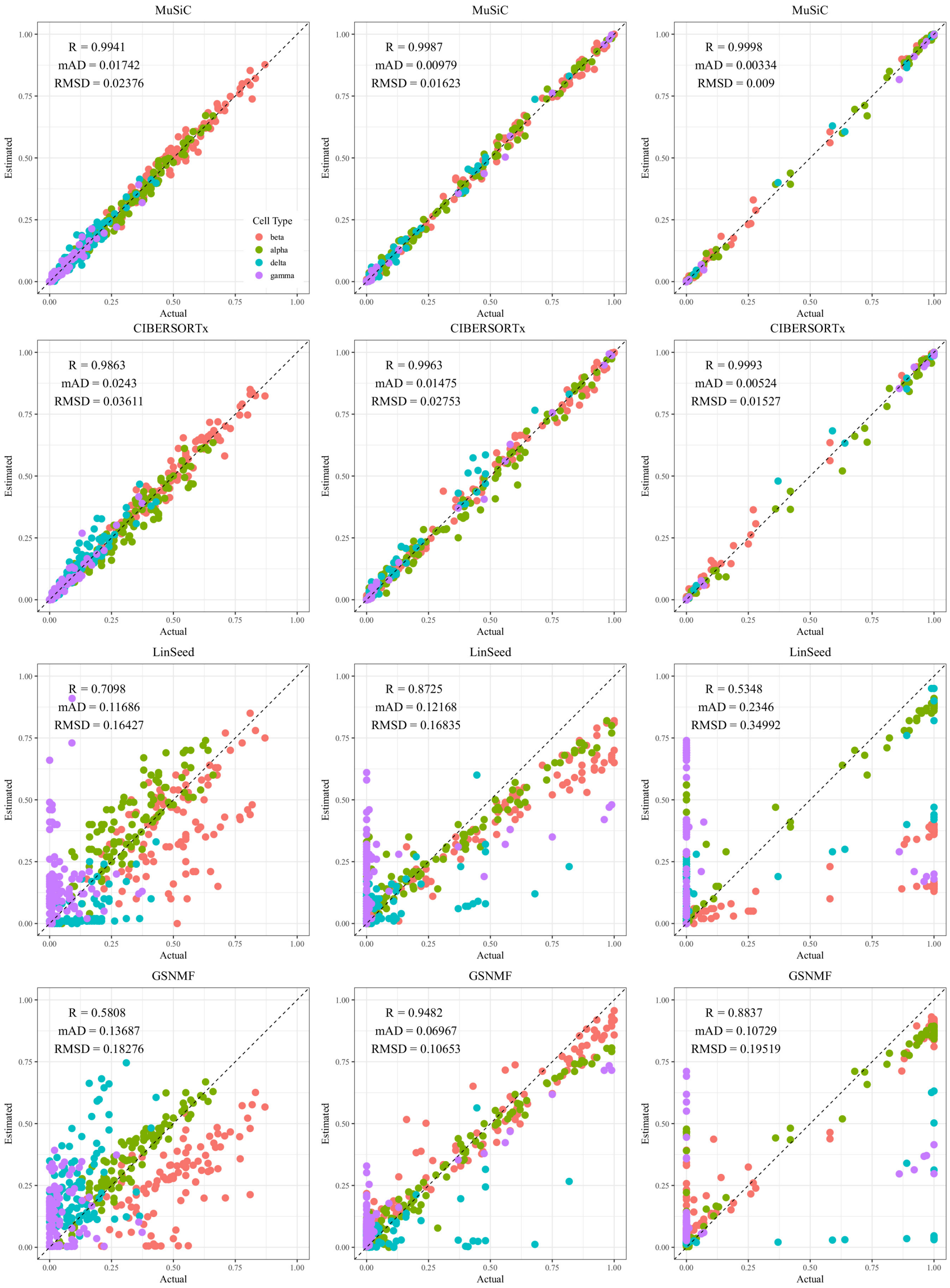

Figure 3: Comparison of estimated vs. actual cell proportions in pseudo-bulk samples from dataset GSE81608. Columns (left to right) represent small, medium, and large cellular composition variation. Rows (top to bottom) shows results from: MuSiC, CIBERSORTx, LinSeed, and GS-NMF. Colors indicate beta (red), alpha (green), delta (cyan), and gamma (purple).

2 Supplementary Figures-Mean Shifting

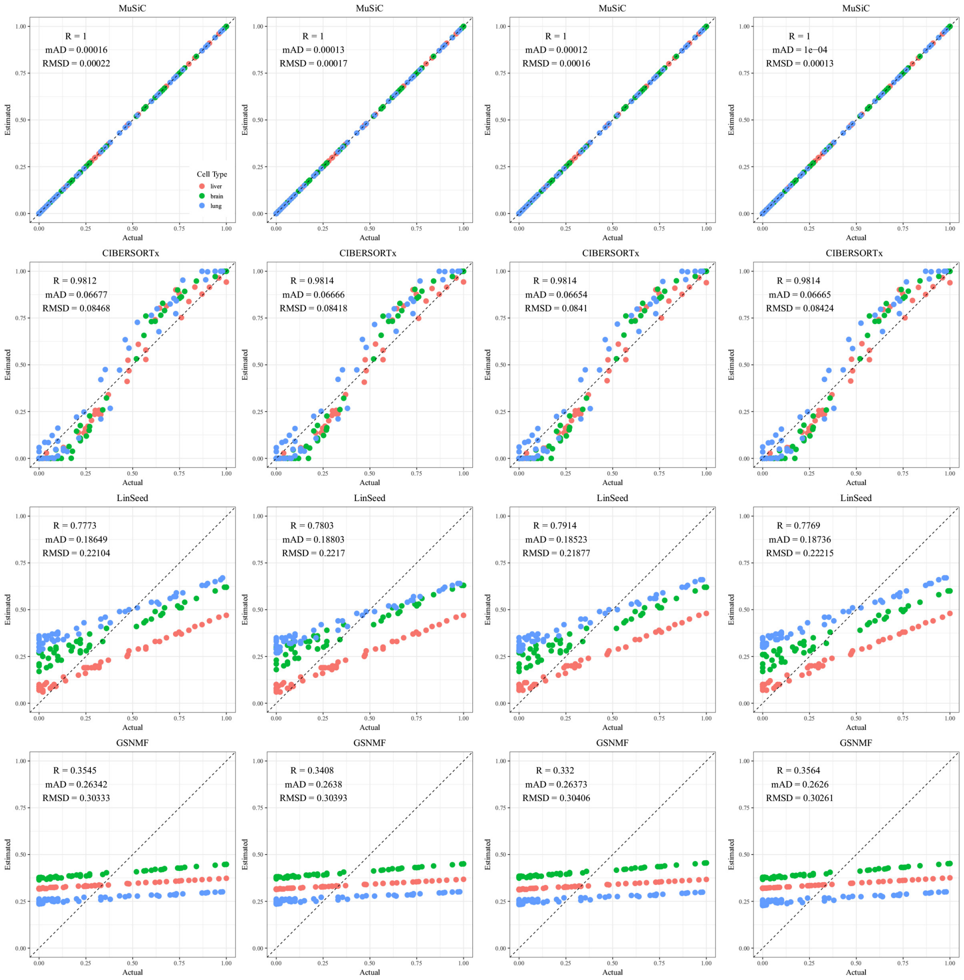

Figure 4: Estimated vs. true cell-type proportions for dataset GSE19830 under mean shifting conditions. Columns (left to right) represent 10%, 30%, 50%, and 70% mean shifts. Rows (top to bottom) are method: MuSiC, CIBERSORTx, Linseed, and GS-NMF. Colors indicate distinct cell types: liver (red), brain (green), and lung (blue).

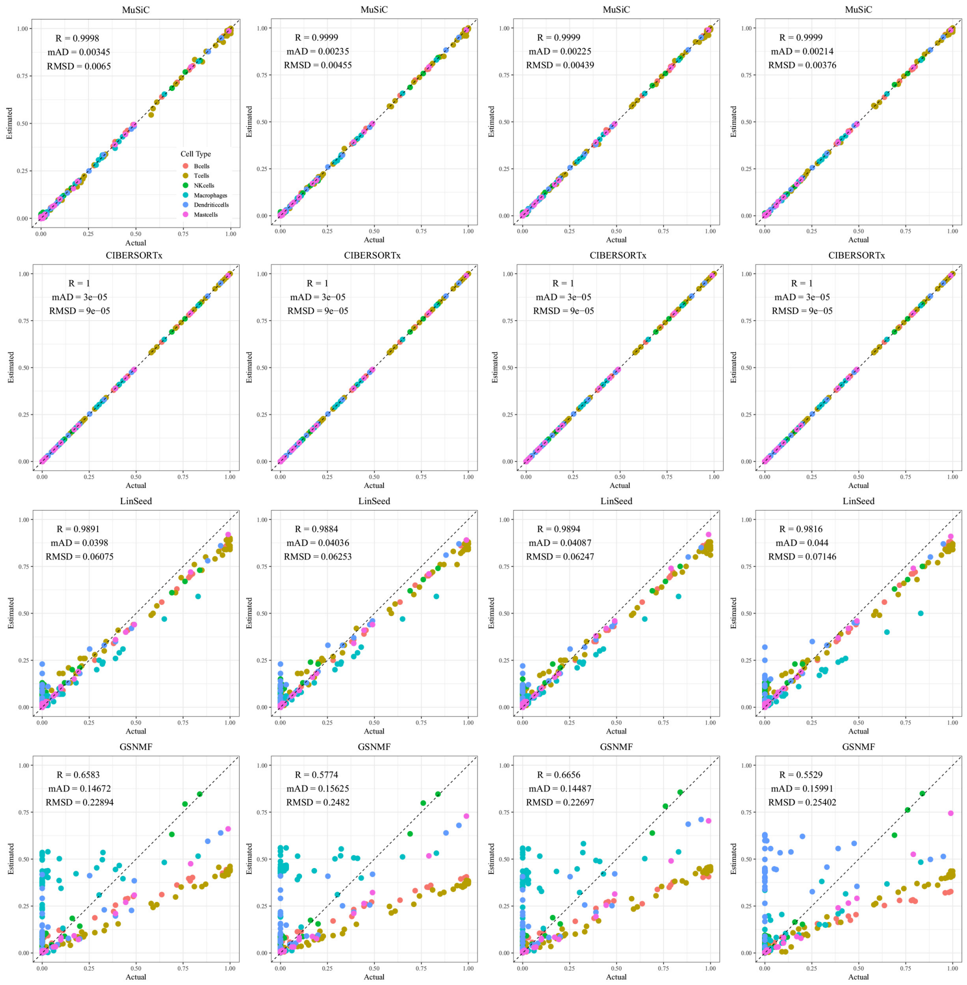

Figure 5: Estimated vs. actual cell-type proportions for dataset LM22 under mean shifting conditions. Columns (left to right) represent 10%, 30%, 50%, and 70% mean shifts. Rows (top to bottom) are method: MuSiC, CIBERSORTx, Linseed, and GS-NMF. Colors represent different cell types: B cells (red), T cells (gold), NK cells (green), Macrophages (cyan), Dendritic cells (blue), and Mast cells (magenta).

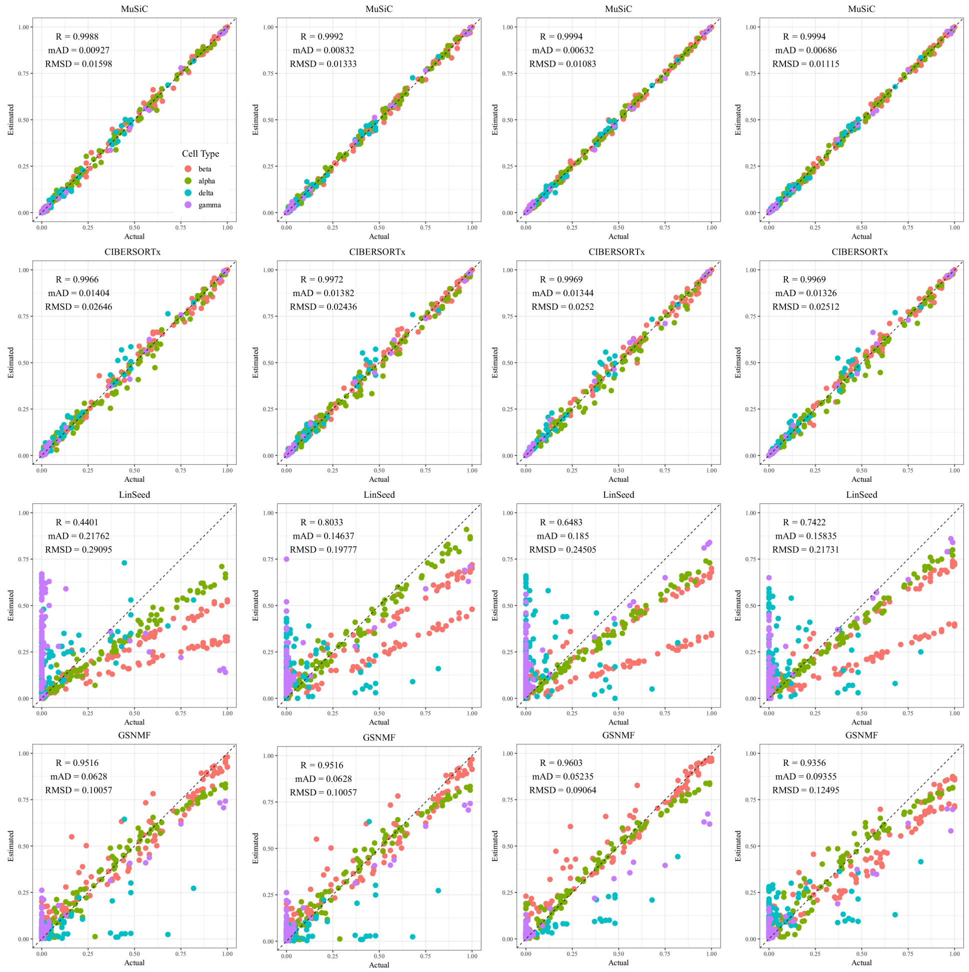

Figure 6: Estimated vs. actual cell-type proportions for dataset GSE81608 under mean shifting conditions. Columns (left to right) represent 10%, 30%, 50%, and 70% mean shifts. Rows (top to bottom) are method: MuSiC, CIBERSORTx, Linseed, and GS-NMF. Colors represent different cell types: beta (red), alpha (green), delta (cyan), and gamma (purple).

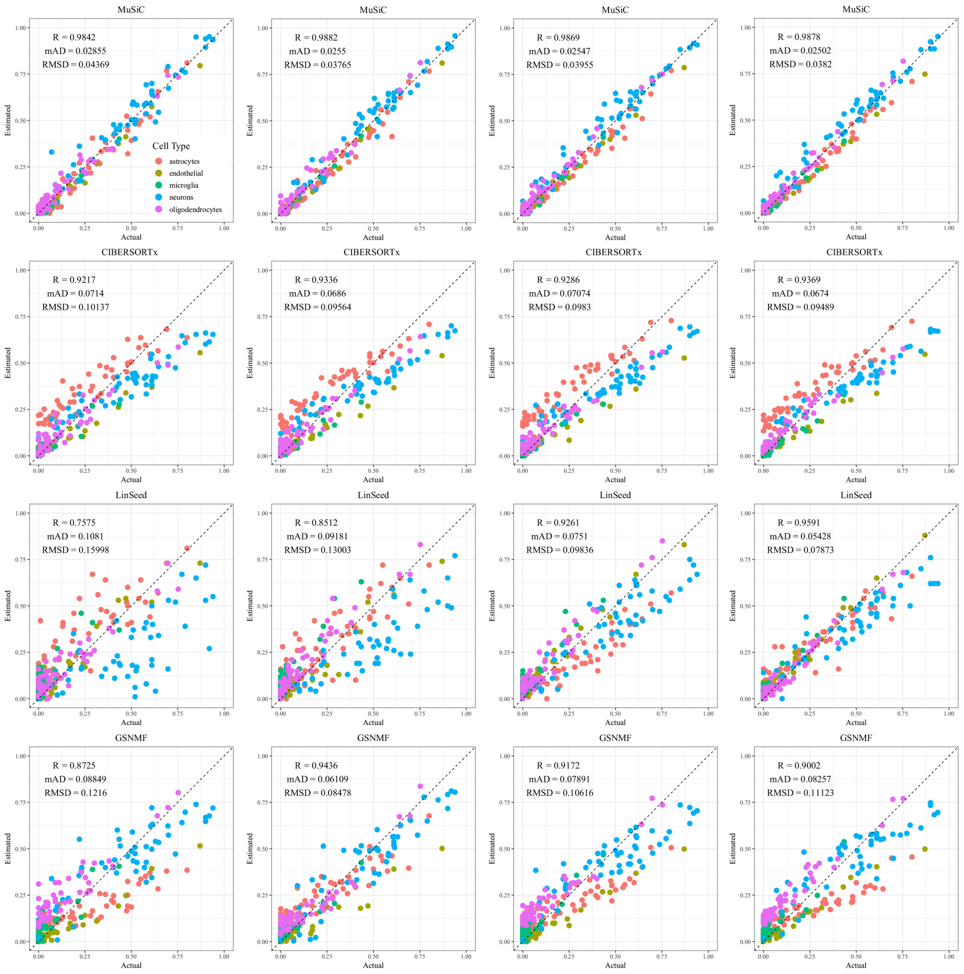

Figure 7: Estimated vs. actual cell-type proportions for dataset GSE67835 under mean shifting conditions. Columns (left to right) represent 10%, 30%, 50%, and 70% mean shifts. Rows (top to bottom) are method: MuSiC, CIBERSORTx, Linseed, and GS-NMF. Colors represent different cell types: astrocytes (red), endothelial (gold), microglia (green), neurons (blue), and oligodendrocytes (magenta).

3 Supplementary Figures-Factoring

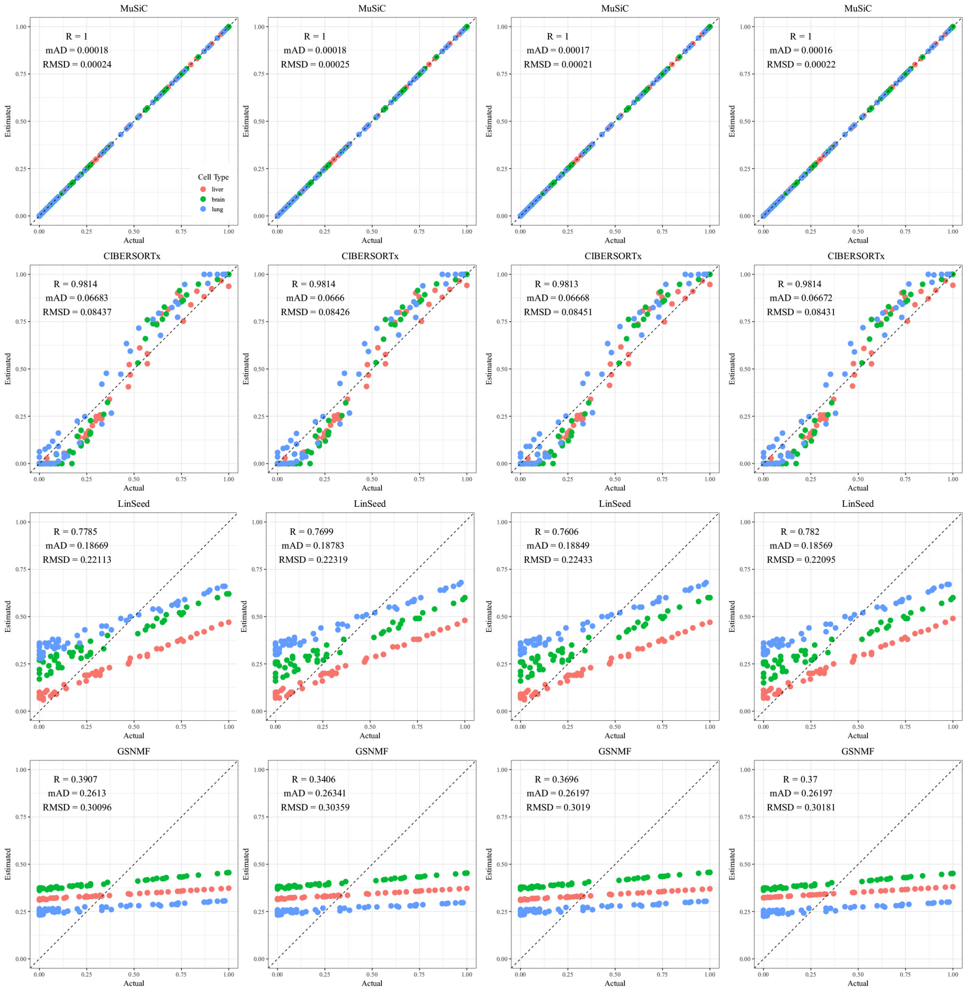

Figure 8: Estimated vs. true cell-type proportions for dataset GSE19830 under factoring scenarios.Columns (left to right) represent factoring values of 1.2, 1.8, 0.8, and 0.4. Rows (top to bottom) indicate deconvolution method: MuSiC, CIBERSORTx, LinSeed, and GS-NMF. Colors indicate distinct cell types: liver (red), brain (green), and lung (blue).

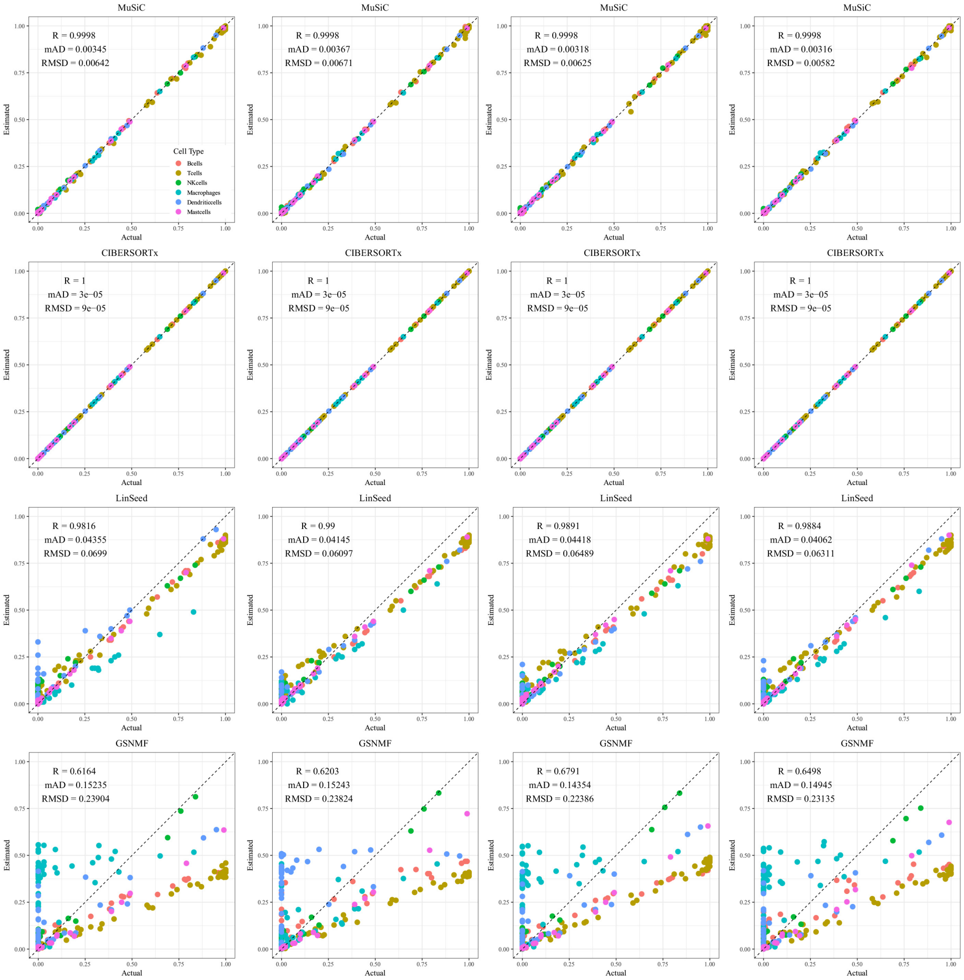

Figure 9: Estimated vs. true cell-type proportions for dataset LM22 under factoring scenarios.Columns (left to right) represent factoring values of 1.2, 1.8, 0.8, and 0.4. Rows (top to bottom) indicate deconvolution method: MuSiC, CIBERSORTx, LinSeed, and GS-NMF. Colors represent different cell types: B cells (red), T cells (gold), NK cells (green), Macrophages (cyan), Dendritic cells (blue), and Mast cells (magenta)).

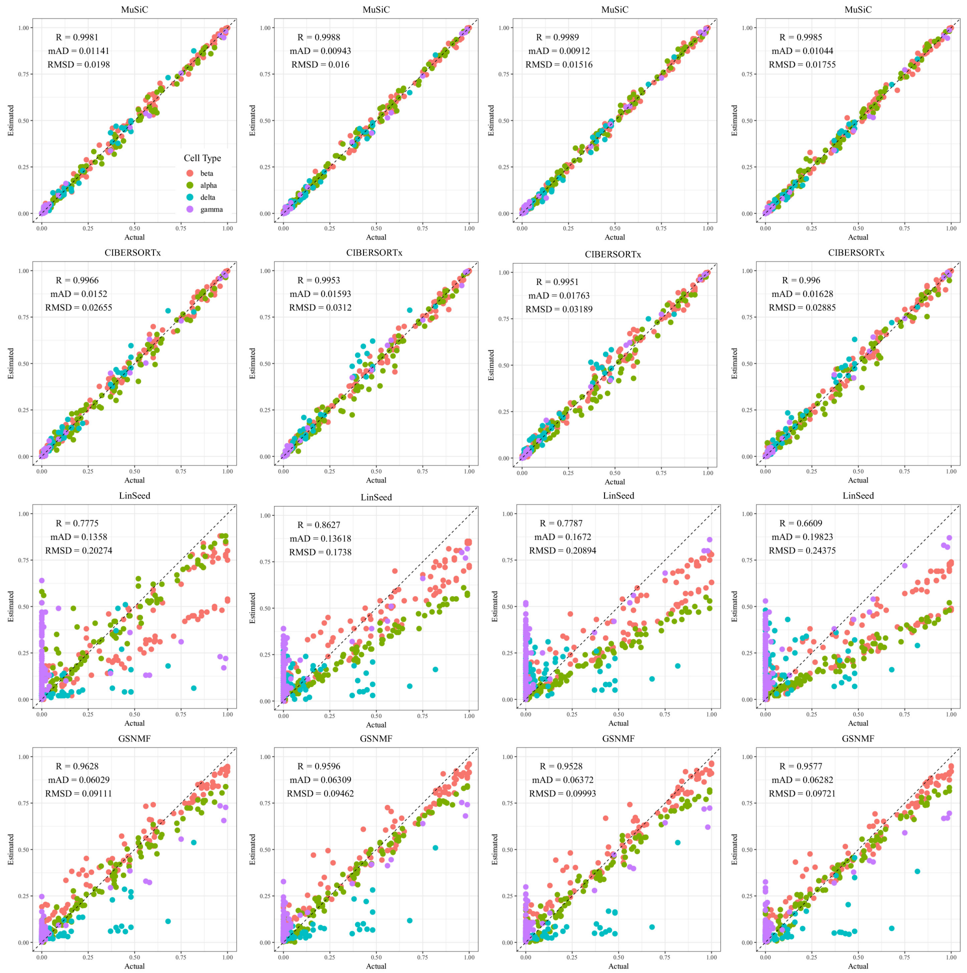

Figure 10: Estimated vs. true cell-type proportions for dataset GSE81608 under factoring scenarios.Columns (left to right) represent factoring values of 1.2, 1.8, 0.8, and 0.4. Rows (top to bottom) indicate deconvolution method: MuSiC, CIBERSORTx, LinSeed, and GS-NMF. Colors represent different cell types: beta (red), alpha (green), delta (cyan), and gamma (purple).

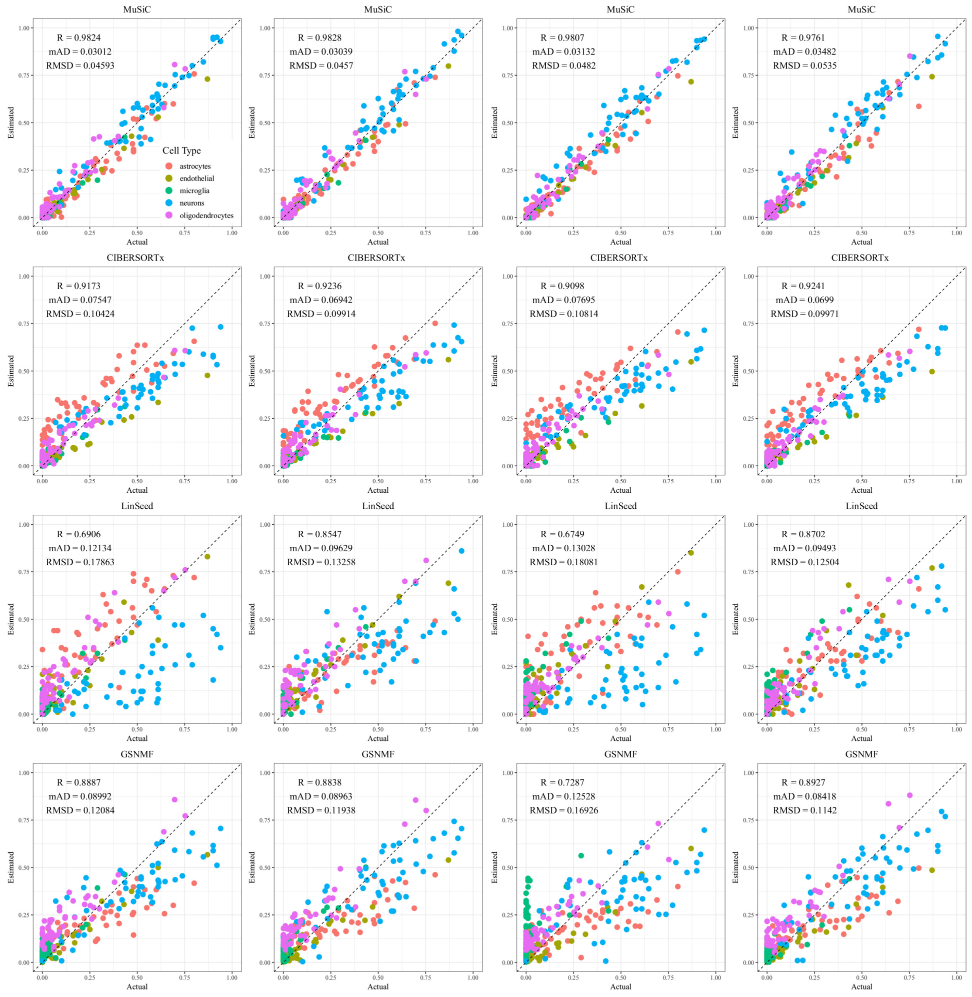

Figure 11: Estimated vs. true cell-type proportions for dataset GSE67835 under factoring scenarios.Columns (left to right) represent factoring values of 1.2, 1.8, 0.8, and 0.4. Rows (top to bottom) indicate deconvolution method: MuSiC, CIBERSORTx, LinSeed, and GS-NMF. Colors represent different cell types: astrocytes (red), endothelial (gold), microglia (green), neurons (blue), and oligodendrocytes (magenta).

4 Supplementary Figures-Truncation

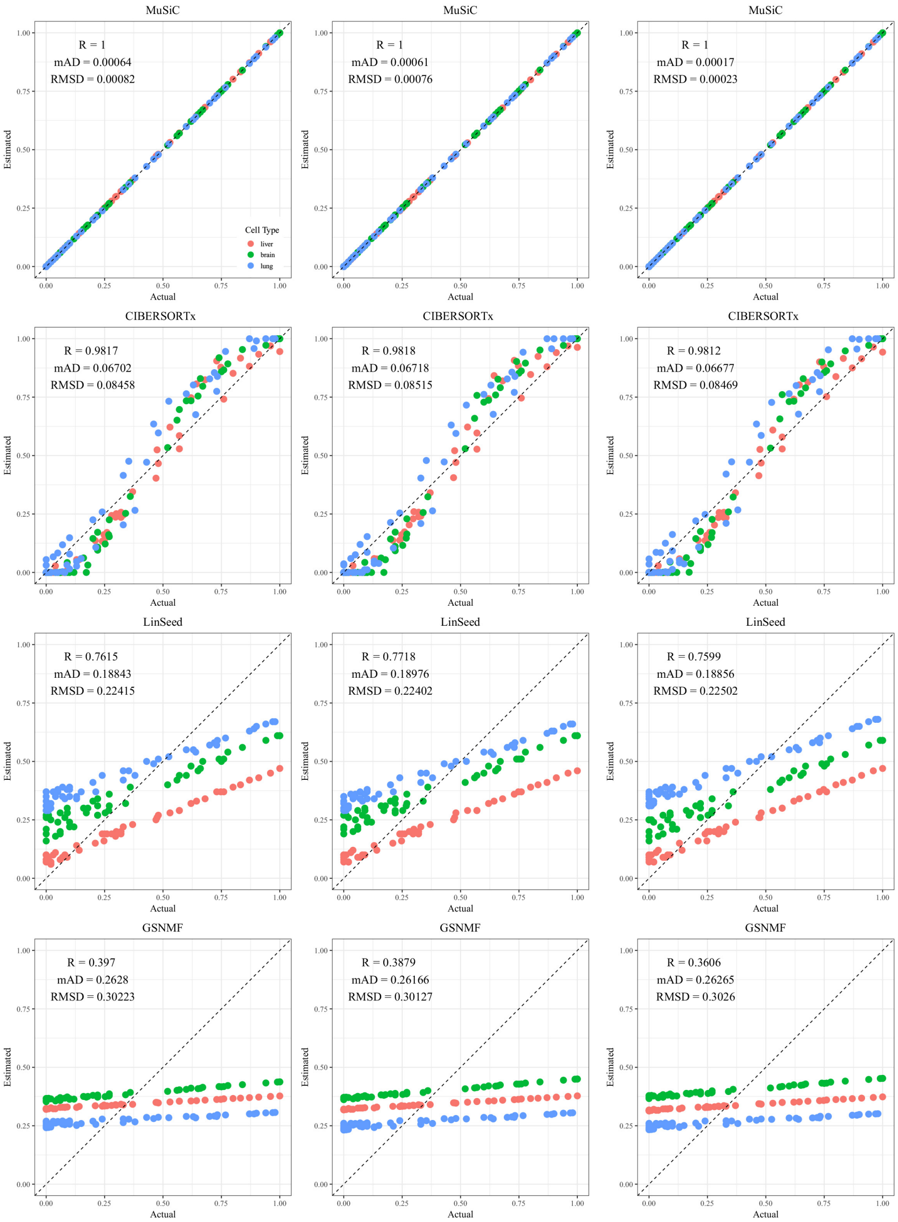

Figure 12: Estimated vs. true cell-type proportions for dataset GSE19830 under different truncation scenarios. Columns (left to right) represent truncation of the top 10%, bottom 10%, and the original dataset. Rows (top to bottom) indicate deconvolution method: MuSiC, CIBERSORTx, LinSeed, and GS-NMF. Colors represent different cell types: liver (red), brain (green), and lung (blue).

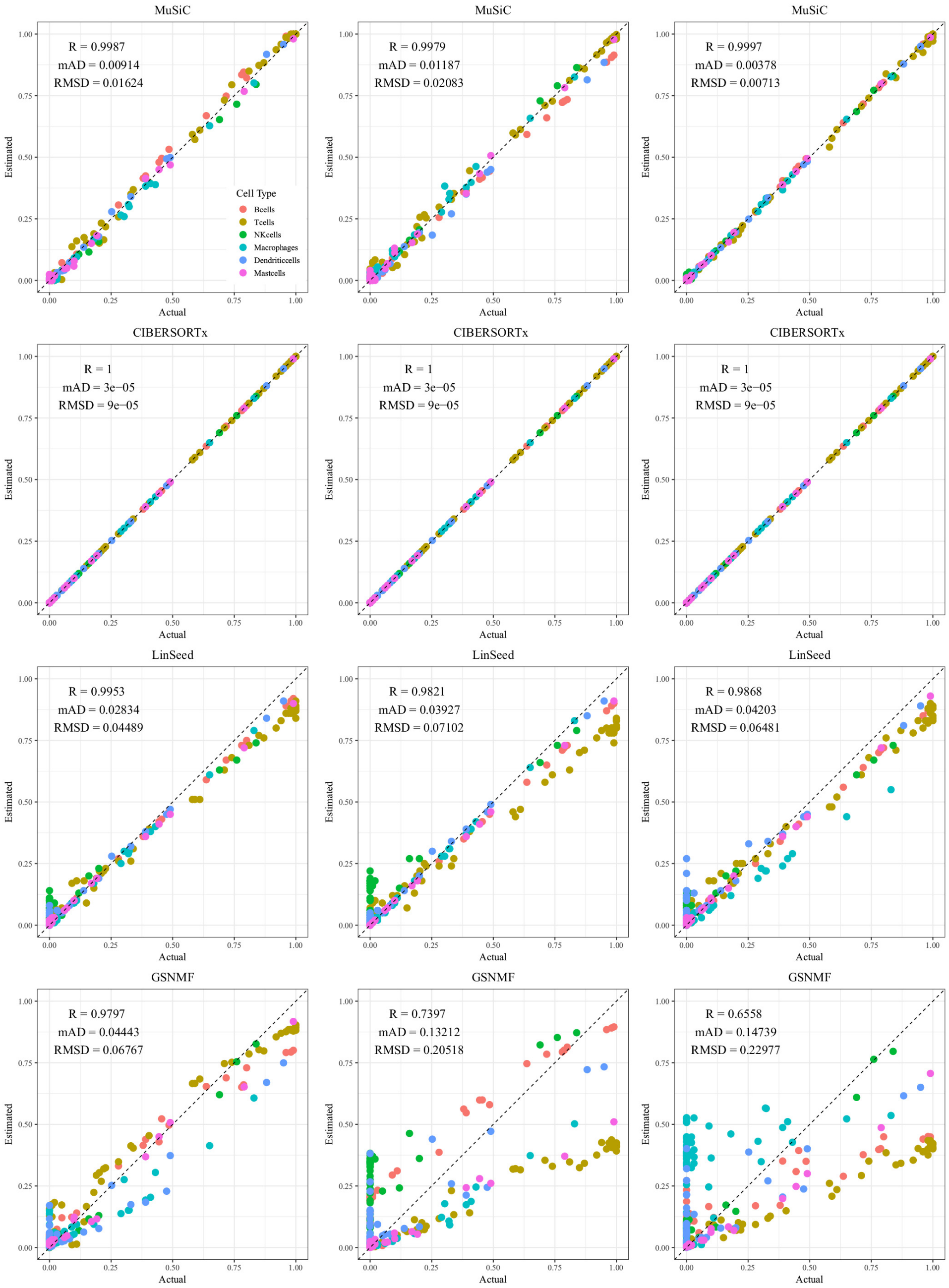

Figure 13: Estimated vs. true cell-type proportions for dataset LM22 under different truncation scenarios. Columns (left to right) represent truncation of the top 10%, bottom 10%, and the original dataset. Rows (top to bottom) indicate deconvolution method: MuSiC, CIBERSORTx, LinSeed, and GS-NMF. Colors represent different cell types: B cells (red), T cells (gold), NK cells (green), Macrophages (cyan), Dendritic cells (blue), and Mast cells (magenta).

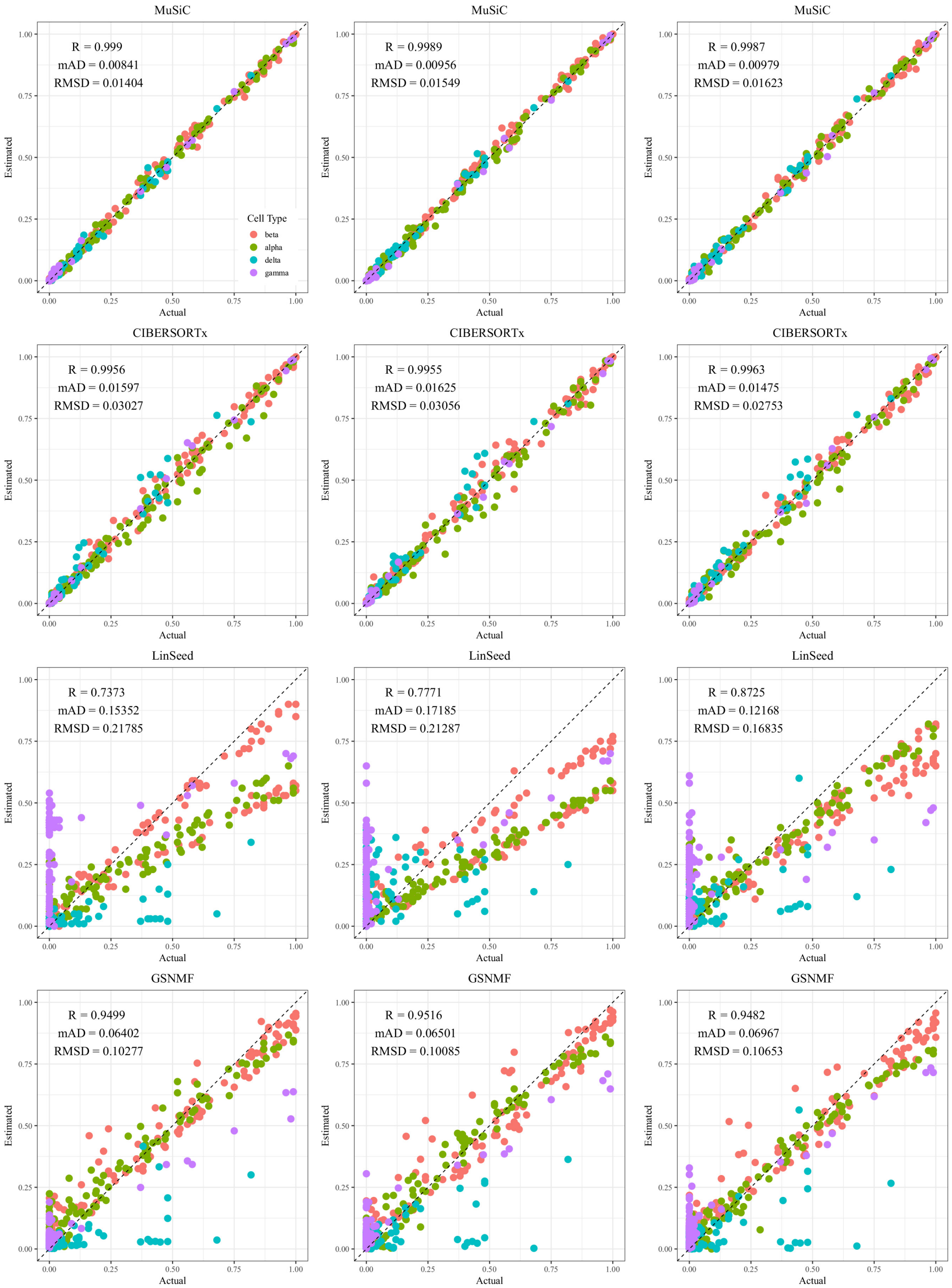

Figure 14: Estimated vs. true cell-type proportions for dataset GSE81608 under different truncation scenarios. Columns (left to right) represent truncation of the top 10%, bottom 10%, and the original dataset. Rows (top to bottom) indicate deconvolution method: MuSiC, CIBERSORTx, LinSeed, and GS-NMF. Colors represent different cell types: beta (red), alpha (green), delta (cyan), and gamma (purple).

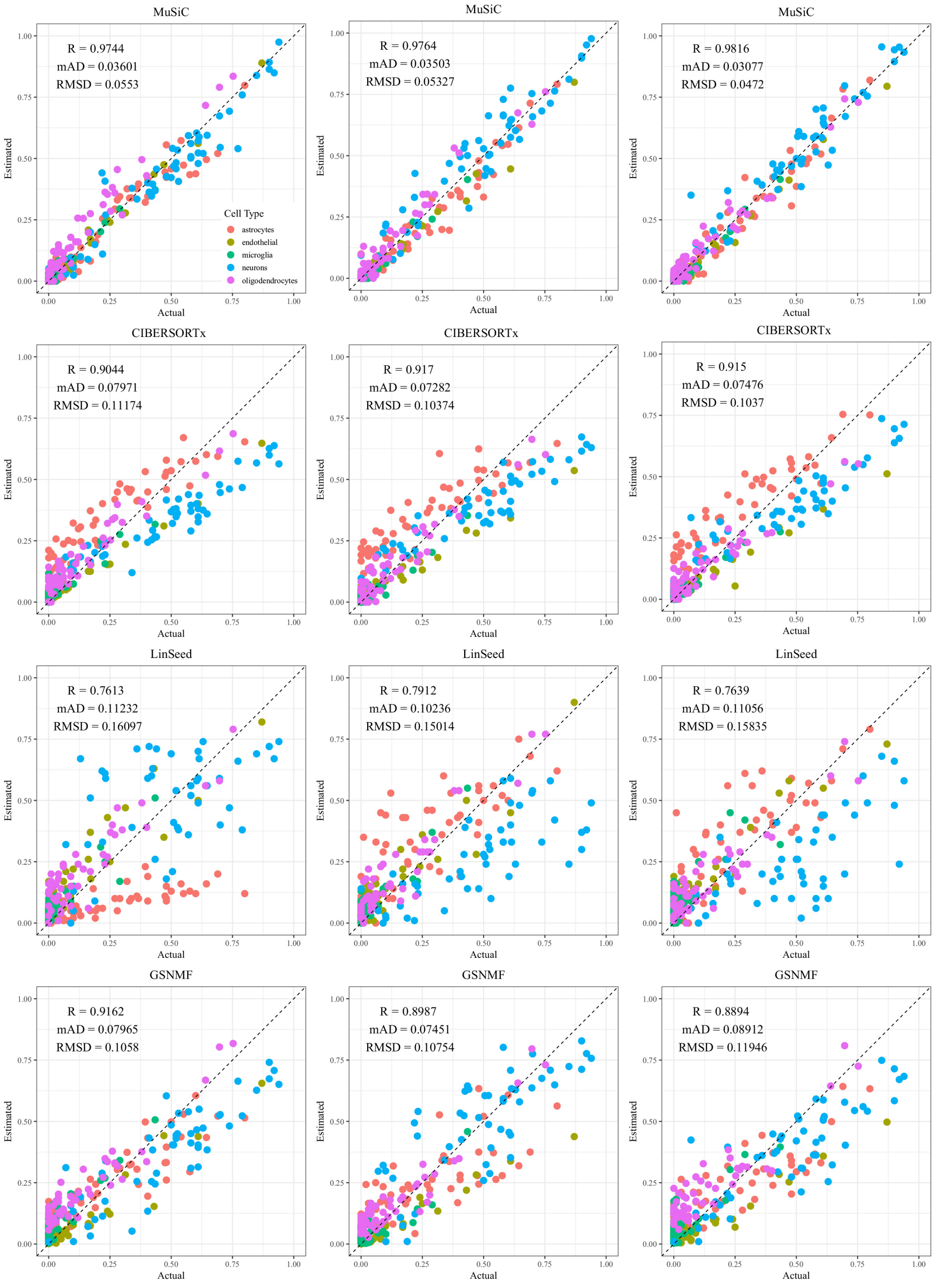

Figure 15: Estimated vs. actual cell-type proportions for dataset GSE67835 under different truncation scenarios.Columns (left to right) represent truncation of the top 10%, bottom 10%, and the original dataset. Rows (top to bottom) indicate deconvolution method: MuSiC, CIBERSORTx, LinSeed, and GS-NMF. Different colors represent different cell types: astrocytes (red), endothelial (gold), microglia (green), neurons (blue), and oligodendrocytes (magenta).
